# Rapid optimization of drug combinations for the optimal angiostatic treatment of cancer

**DOI:** 10.1007/s10456-015-9462-9

**Published:** 2015-04-01

**Authors:** Andrea Weiss, Xianting Ding, Judy R. van Beijnum, Ieong Wong, Tse J. Wong, Robert H. Berndsen, Olivier Dormond, Marchien Dallinga, Li Shen, Reinier O. Schlingemann, Roberto Pili, Chih-Ming Ho, Paul J. Dyson, Hubert van den Bergh, Arjan W. Griffioen, Patrycja Nowak-Sliwinska

**Affiliations:** 1 Institute of Chemical Sciences and Engineering, Swiss Federal Institute of Technology (EPFL), 1015 Lausanne, Switzerland; 2 Department of Medical Oncology, VU University Medical Center, 1007 MB Amsterdam, The Netherlands; 3 Med-X Research Institute, School of Biomedical Engineering, Shanghai Jiao Tong University, Shanghai, 200030 China; 4 Department of Mechanical and Aerospace Engineering, University of California, Los Angeles, CA 90095 USA; 5 Department of Visceral Surgery, Centre Hospitalier Universitaire Vaudois, 1011 Lausanne, Switzerland; 6 Ocular Angiogenesis Group, Departments of Ophthalmology and Cell Biology and Histology, Academic Medical Center, 1100 DD Amsterdam, The Netherlands; 7 Department of Medicine, Roswell Park Cancer Institute, Buffalo, NY 14263 USA

**Keywords:** Anti-angiogenesis, Combination therapy, Drug–drug interactions, Feedback system control, Search algorithm

## Abstract

Drug combinations can improve angiostatic cancer treatment efficacy and enable the reduction of side effects and drug resistance. Combining drugs is non-trivial due to the high number of possibilities. We applied a feedback system control (FSC) technique with a population-based stochastic search algorithm to navigate through the large parametric space of nine angiostatic drugs at four concentrations to identify optimal low-dose drug combinations. This implied an iterative approach of in vitro testing of endothelial cell viability and algorithm-based analysis. The optimal synergistic drug combination, containing erlotinib, BEZ-235 and RAPTA-C, was reached in a small number of iterations. Final drug combinations showed enhanced endothelial cell specificity and synergistically inhibited proliferation (*p* < 0.001), but not migration of endothelial cells, and forced enhanced numbers of endothelial cells to undergo apoptosis (*p* < 0.01). Successful translation of this drug combination was achieved in two preclinical in vivo tumor models. Tumor growth was inhibited synergistically and significantly (*p* < 0.05 and *p* < 0.01, respectively) using reduced drug doses as compared to optimal single-drug concentrations. At the applied conditions, single-drug monotherapies had no or negligible activity in these models. We suggest that FSC can be used for rapid identification of effective, reduced dose, multi-drug combinations for the treatment of cancer and other diseases.

**Electronic supplementary material:**

The online version of this article (doi:10.1007/s10456-015-9462-9) contains supplementary material, which is available to authorized users.

## Introduction

Anti-angiogenic therapies are routinely used in the treatment of various cancers [1–3]. Their contribution to the prolongation of patient survival, however, is often limited mainly due to disease and patient heterogeneity [4, 5], toxicity [6], induction of metastasis [7] and drug resistance [8, 9]. Redundancy of growth factor signaling pathways makes angiogenesis a robust physiological function [10, 11], where targeting multiple pathways with drug combinations may be necessary for efficient therapy [12]. Although difficult to predict, in such drug combinations one may encounter synergistic, additive or antagonistic interactions between drugs. Synergistic interactions can lead to effective angiogenesis inhibition at reduced doses as compared to single-drug therapies. Combination strategies may thus lead to enhanced efficacy [13, 14] with limited side effects [15] and reduced probability of developing drug resistance [16, 17].

Combinations of anti-angiogenic drugs have often resulted in significant clinical toxicity [18], even when designed to target complementary pathways [19]. This is because drugs to be combined are frequently selected based on their success as single agents [20] and tend to be used in combination at their maximum tolerated single agent doses, thus increasing the risk of toxicity and resistance [21]. When trying to identify an optimal combination starting from, for instance, 10 drugs at 5 doses, one will have to test nearly 10 million (5^10^) combinations. To overcome this challenge, we employed a feedback system control (FSC) technique to rapidly identify the most powerful drug combinations with minimal experimental effort [22] (Supplementary Methods). In combination with the differential evolution (DE) algorithm [23], an iterative approach of experimental testing in an endothelial cell viability assay and mathematical analysis (a process of selection, where only the permutations which improve the system’s response are maintained) drove the system to converge toward an optimal solution, i.e., maximal inhibition of endothelial cell growth. Although others have tried to optimize drug combinations [24–27], see Supplementary Methods, the advantage of our approach is that FSC is phenotypically driven, i.e., no mechanistic information is required in order to rapidly identify experimentally verifiable optimal drug combinations [22].

The aim of the present study was to find an optimal low-dose, synergistic anti-angiogenic drug combination using the FSC technology, and to validate this drug combination in preclinical tumor models. The FSC technique, together with a second-order linear regression model to allow for elimination of less effective drugs, resulted in the identification of the optimal low-dose combination containing erlotinib (EGFR inhibitor [28]), RAPTA-C (histone inactivator [29]) and BEZ-235 (a dual PI3K/mTOR inhibitor [30]). This final drug combination synergistically inhibited ECRF24 viability, while having minimal effects on non-endothelial cell types. We successfully translated this in vitro optimized drug combination to inhibit tumor growth in two preclinical tumor models.

## Materials and methods

### Cell viability, migration and apoptosis assay

Cell viability and migration assays were performed as previously described. Cells were seeded in a 96-well culture plate at a density of 2.5–10 × 10^3^ cells/well. Cells were incubated with drugs for 72 h (for drug acquisition and cells and culture conditions, see Supplementary Methods). Drugs were premixed in culture medium and applied at the doses provided in Table 1. Cell viability was assessed using the CellTiter-Glo luminescence assay (Promega, Madison, WI, USA). For migration assays, ECRF24 and 786-O were seeded in 96-well cell culture plates (3 × 10^4^ cells/well) 24 h prior to making the scratch (Peira Scientific Instruments, Beerse, Belgium). Drugs were premixed in culture medium and applied at doses indicated in Supplementary Fig. S2A. Images were automatically captured on a Leica DMI3000 microscope (Leica, Rijswijk, Netherlands) at 5× magnification with Universal Grab 6.3 software (DCILabs, Keerbergen, Belgium). Scratch sizes were determined at *t* = 0 h and *t* = 7 h using Scratch Assay 6.2 (DCILabs), and values reported represented the absolute closure of the scratch (initially subtracting the final scratch area). Apoptosis was measured after drug exposure, trypsinization and incubation with propidium iodide (PI, 20 µg/ml) in DNA extraction buffer [31], by flow cytometry. Tip cells were flow cytometrically quantified by CD34 [32] staining, and morphology was studied in vivo using the CAM assay [33] (see Supplementary Methods).

**Table 1:** Drug dose values used in the in vitro cell viability assays

	Dose (μM)			
	Drug	3 (ED_10_)^a^	2 (ED_5_)^b^	1 (ED_0_)^c^
1.	Anginex	1.80^d^	0.76	0.13
2.	Bevacizumab	15.00	10.00	1.00
3.	Axitinib	1.00	0.30	0.01
4.	Erlotinib	2.00	0.50	0.10
5.	Anti-HMGB1 Ab	0.17	0.09	0.02
6.	Sunitinib	0.50	0.10	0.05
7.	Anti-vimentin Ab	0.26	0.17	0.09
8.	RAPTA-C	5.00	1.00	0.05
9.	BEZ-235	0.005	0.001	0.0005

^a^Dose 3, representing ED_10_, is the dose where 10 % of the maximal response was observed

^b^Dose 2, representing ED_5_, is the dose where 5 % of the maximal response was observed

^c^Dose 1, representing ED_0_, is the dose representing half the maximal concentration where no effect was observed

^d^Concentrations throughout the table are in μM

### The feedback system control (FSC) technique and data modeling

The FSC technique was employed as previously described [34, 35]. FSC was implemented using the DE algorithm, and two separate optimizations were performed with the cellular outputs of ECRF24 cell viability (proliferation) and migration assays. Nineteen drug combinations were tested per iteration, and 10 iterations were performed in each optimization until a plateau in the best output value was reached. For dilutions and culture conditions, see Supplementary Methods. The cells were incubated in 50 µl of each combination for 72 h in the viability assay or for 7 h in the migration assay.

Second-order linear regression models were generated using the data obtained from each optimization. Data were modeled using real concentration values, and both concentration values and cell viability output data were transformed using the *z* score function in MATLAB. For detailed description, see Supplementary Methods.

### Human ovarian carcinoma grown on the chicken chorioallantoic membrane (CAM)

Human ovarian carcinoma tumors were implanted on the CAM as previously described [36]. On embryo development day (EDD) 7, 1 × 10^6^ A2780 carcinoma cells were prepared as a spheroid in a 25-µl hanging drop and were transplanted onto the CAM surface 3 h after preparation. Treatment began 3 days after tumor implantation (EDD10) when vascularized tumors were visible. Drug combinations were freshly prepared and administered as a 20-µl intravenous injection. Treatment was performed twice, and tumor growth was monitored and measured daily (volume = width^2^ × length × 0.52).

### Colorectal carcinoma xenograft model

Female Swiss nu/nu mice aged 6–8 weeks were obtained from Charles River (weight 20–30 g). Mice were inoculated in the right flank with 100 µl DMEM with 1 million LS174T cells. LS174T cells were obtained from ECACC, Salisbury, UK (authentication by STR PCR), and were used within 6 months of resuscitation. Palpable tumors were present within 3–5 days, at which time treatment was initiated. Mice were treated daily by oral gavage and i.p. injection as indicated (Table 2) and were monitored daily for tumor size and body weight (see Supplementary Methods).

**Table 2:** Drug dose values used for in vivo assays

Treatment^b^	Compounds^a^				% CTRL ± SEM^c^
	3 (axitinib)	4 (erlotinib)	8 (RAPTA-C)	9 (BEZ-235)	
*CAM (µg/kg)*					
I	0	29	615	0.04	41 ± 9.0
II	0	29	307	0.04	51 ± 14
VI	0	2.9	307	0.04	32 ± 4.0
VII	18	29	230	0.02	13 ± 6.0
VIII	0	29	307	0	47 ± 14
*Mice (mg/kg)*					
VI	0	15	40	10	24 ± 14
VIII	0	5	40	0	84 ± 16
4_1_	0	5	0	0	102 ± 25
4_2_	0	15	0	0	94 ± 34
4_opt_	0	50	0	0	29 ± 9.0
8_2_	0	0	40	0	90 ± 17
8_opt_	0	0	100	0	58 ± 9.0
9_4_	0	0	0	10	77 ± 12
9_opt_	0	0	0	30	33 ± 14

^a^Corresponding dose of each compound for single-drug and combination therapy. Compounds are represented as numbers **3**, **4**, **8** or **9**, representing axitinib, erlotinib, RAPTA-C or BEZ-235, respectively. Drug doses are provided in µg/kg for the CAM model and mg/kg in the mouse model

^b^Administered treatment, either a single drug represented by the drug number (**3**, **4**, **8**, **9**) and the dosage level indicated as a subscript, or drug combinations represented by the letters **I**–**VIII**

^c^The respective tumor growth inhibition efficacy represented as a percentage of the control (±SEM)

### Immunohistochemistry

CD31 staining (SZ31, Dianova, Hamburg, Germany) was performed using donkey anti-rat biotinylated secondary antibodies (Jackson, Suffolk, UK) and streptavidin-HRP (Dako, Glostrup, Denmark) and visualized by 3,3′-diaminobenzidine (DAB, see Supplementary Methods).

### Statistical analysis

Values are given as mean values ± SD. Statistical analysis was performed using a two-sided student’s *t* test and the two-way ANOVA assay. *p < 0.05 and **p < 0.01 were considered statistically significant.

## Results

### Selection of drug combinations by the FSC technique

**Fig. 1:**
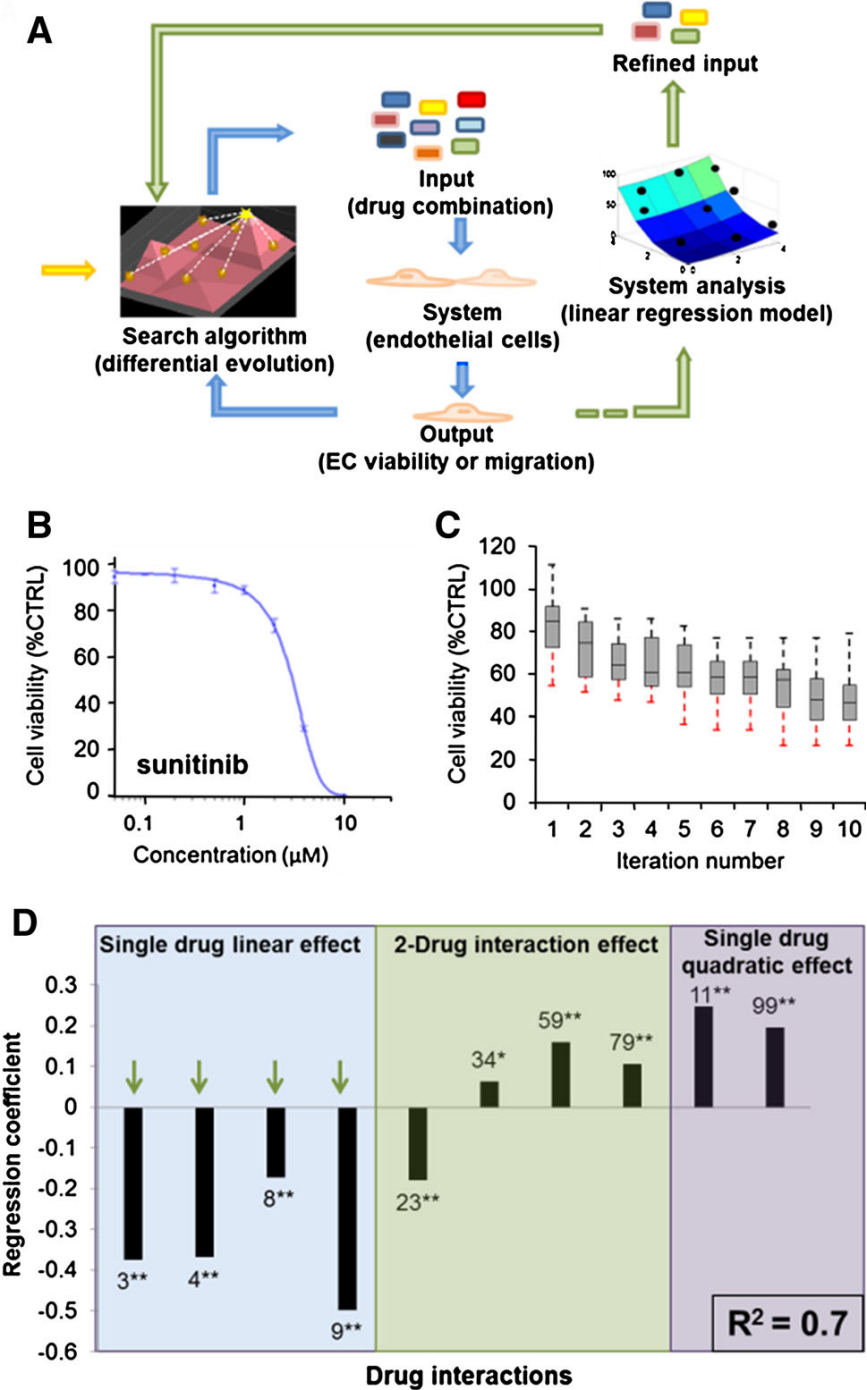
Optimization of the inhibition of endothelial cell viability. **a** Schematic diagram of the FSC technique loop (*blue arrow* loop) and modeling (*green arrow* loop) used for in vitro drug optimization. FSC starts with randomly selected drug combinations (*yellow arrow*) and implements an algorithm-guided closed-loop feedback search to iteratively optimize the results of an in vitro cell assay (*blue arrows*). Once a plateau in the output is reached, the data obtained from the optimization are used to model the system, analyze drug interactions and eliminate certain drugs (*green arrows*). Using a refined set of drugs, the drug combination is again optimized with FSC (*blue arrows*). **b** Dose–response curve of sunitinib for cell viability bioassay. **c** Output results (in vitro EC viability, represented as a percentage of the control) for the 10 iterations of the FSC optimization performed. *Box plots* provide median and interquartile ranges of the cell response to the 19 best drug combinations identified by the end of each iterative cycle of the FSC optimization. *Dotted lines*, representing maximum and minimum (*red*) output values, showed no improvement in the best-optimized combination over iterations 8–10. **d** Regression coefficients obtained from the stepwise linear regression model generated with the data obtained from the optimization described above. The coefficient of determination (*R* ^2^) is provided in the bottom right of the graph. *Green arrows* indicate single-drug contributions which significantly inhibit EC viability. * indicates *p* value <0.05 and ** indicates *p* value <0.01. (Color figure online)

Nine drugs targeting a broad spectrum of endothelial cell signaling pathways (Supplementary Fig. S1; Supplementary Methods) were selected for FSC-based screening (Fig. 1a): anginex (**1**), bevacizumab (**2**), axitinib (**3**), erlotinib (**4**), anti-HMGB1 Ab (**5**), sunitinib (**6**), anti-vimentin Ab (**7**), RAPTA-C (**8**) and BEZ-235 (**9**). Single-drug dose–response curves were generated for both cell viability (example for sunitinib provided, Fig. 1b) and migration, using in vitro bioassays (Supplementary Fig. S2A). The optimization was carried out with each compound at four low doses. The highest concentration, dose 3 or ED_10_, was the dose where 10 % of the maximal response was observed, dose 2 or ED_5_, where 5 % of the maximal response was observed, dose 1 or ED_0_, represented half the maximal dose where no effect was observed, and dose 0, where no drug was present (Table 1). Starting from randomly selected drug combinations (Fig. 1a, yellow arrow), the FSC technique implements an algorithm-guided closed-loop feedback search to iteratively optimize the results of an in vitro cell assay (blue arrows). The box plot in Fig. 1c provides the median and interquartile ranges of the output results of the drug combinations identified by the end of each iterative cycle of the FSC optimization. After 10 iterations of 19 drug mixtures, the optimization goal was reached, i.e., no further improvement of the lowest output efficacy could be achieved, indicating that the maximum activity (approx. 70 % inhibition) had been reached.

The data obtained from this optimization process were used to build a second-order stepwise linear regression model [37] (Supplementary Methods) to determine the relative importance of the individual drugs. This model generated regression coefficients (Fig. 1d) corresponding to single-drug linear effects (left panel), two-drug pair-wise interaction effects (middle panel) and single-drug quadratic effects (right panel). Compounds with the largest negative regression coefficients, i.e., axitinib, erlotinib, RAPTA-C and BEZ-235, inhibited ECRF24 viability most effectively (Fig. 1d, green arrows). A regression model containing all regression coefficients (i.e., a non-stepwise linear regression model) is provided in Supplementary Fig. S2B.

In a parallel approach, we also investigated the best drug combinations for ECRF24 migration inhibition. Even though single drugs generally showed a stronger response in the cell migration assay (Supplementary Fig. S2A), the process of migration was less affected, reaching a maximum effect of 40 % inhibition in the given conditions (Supplementary Fig. S3B). The optimization of EC migration inhibition was not further pursued. Yet, regression analysis also revealed strong single-drug linear and quadratic contributions for erlotinib, RAPTA-C and BEZ-235.

### Refined search leads to further optimized synergistic drug combinations

**Fig. 2:**
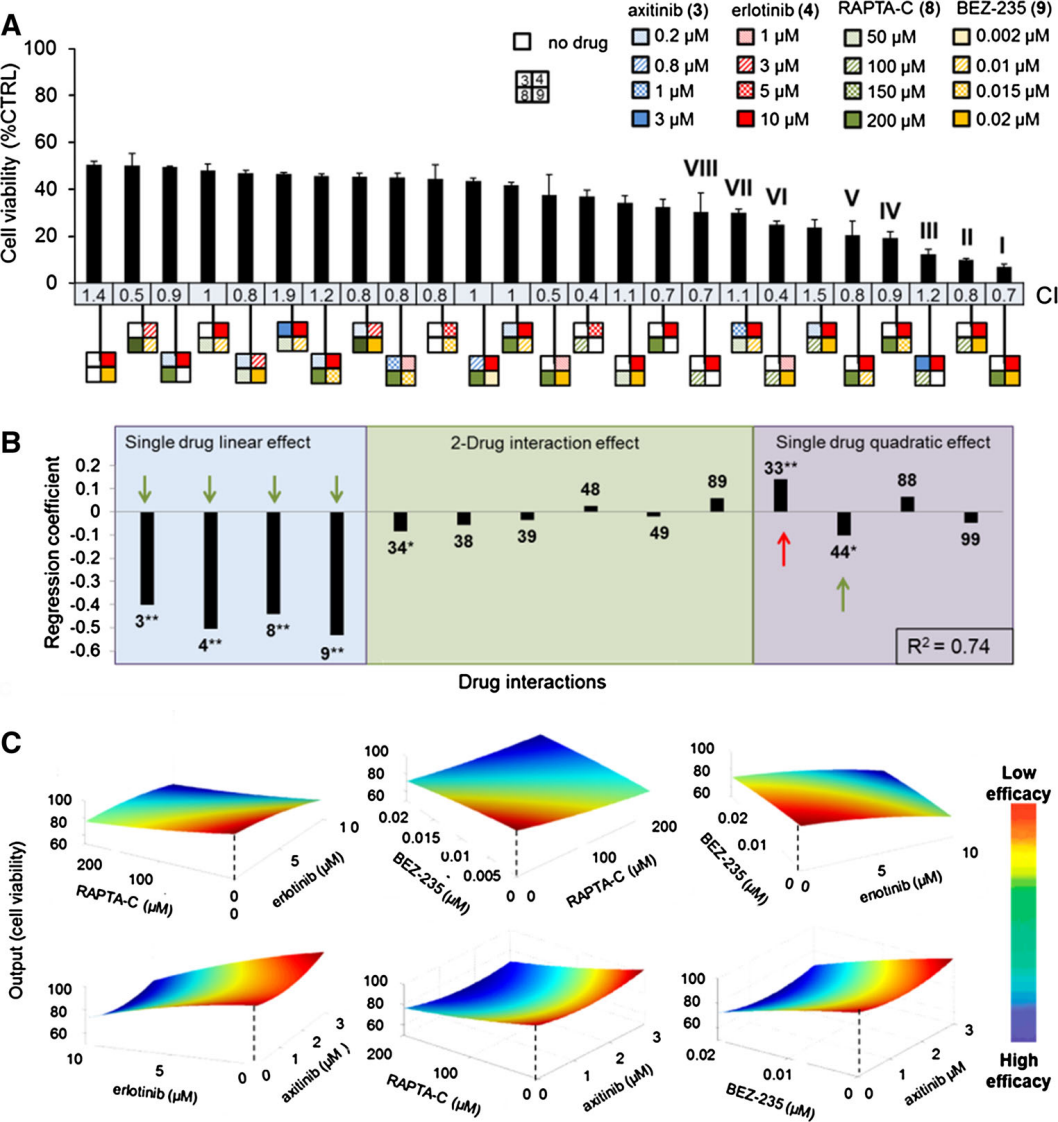
Identification of the optimal four-drug combinations for the inhibition of ECRF24 viability. **a** Efficacy of the best combinations identified to inhibit ECRF24 viability, using the concentrations of each drug presented in the legend at the *top right*. Best-performing combinations resulting in 50 % or more inhibition are shown, with their corresponding combination index (CI) values calculated using CompuSYN^®^, indicating synergistic (CI < 1), additive (CI = 1) or antagonistic (CI > 1) interactions. The square icons present the specific combinations, where each position in the square and color corresponds to a specific drug [i.e., axitinib (**3**) in *blue*, erlotinib (**4**) in *red*, RAPTA-C (**8**) in *green* and BEZ-235 (**3**) in *yellow*] and the concentrations (in µM) of each compound are represented by the different patterns. The most promising combinations, labeled **I**–**VIII**, represent a mean of at least two independent experiments, with three replications each, and *error bars* represent the SEM. **b** Regression coefficients for the second-order linear regression model generated based on the data from the optimization of the refined four-drug combination. The *green arrows* indicate significant regression terms that inhibit cell viability, while the *red arrow* indicates terms that stimulates cell activity. **c** Response surfaces show the effect on the system output of varying the concentration of two drugs, while the concentration of the other two drugs remains fixed. Note the smoothness of the curves, indicating that moderate changes in the dosing of a single drug do not result in major output differences. * indicates significance *p* value <0.05 and **p value <0.01. (Color figure online)

Subsequently, a second FSC-based optimization was performed with the above-selected compounds, i.e., axitinib (**3**), erlotinib (**4**), RAPTA-C (**8**) and BEZ-235 (**9**), each now considered at five drug doses with a maximum activity of 25 % at the highest dose (Fig. 2a; single-drug effects in Supplementary Fig. S3). The most effective combinations resulting in more than 50 % inhibition of ECRF24 cell viability identified in the second screen are provided in Fig. 2a. The square icons represent the individual drug combinations, where the color and pattern can be used to identify the drug and its applied dosage. The strongest synergistic activity [i.e., combination index (CI) <1] was observed for combinations containing **4**+**8**+**9** (combinations labeled **I**, **II**, **IV**, **V**, **VI**, Fig. 2a (remaining results in Supplementary Fig. S4) or only **4**+**8** (labeled **VIII**). Two of the effective combinations identified, **III** and **VII**, showed antagonism (CI > 1), and both contained axitinib (**3**).

Linear regression modeling of data showed the single-drug linear contributions of all compounds, as well as the single-drug quadratic effect of **4**, to be significant (Fig. 2b, green arrows). Response surfaces (Fig. 2c) provide a visual representation of the relationship between the system output (EC viability) and the varying dose of only two drugs in the combination. Interestingly, surfaces containing **3** (bottom row) show that increasing the dose of **3** does not enhance the combination efficacy (red). These response surfaces show a relatively smooth response when doses of the given drugs are varied. This “smoothness” indicates that a moderate change in the dose of a single drug in the range of the experimental conditions near the optimal output investigated will most likely not result in a significant change in the output response. This implies a certain amount of “stability” in the optimal drug mixture which may facilitate its translation to different models.

### Selected optimized drug combinations exhibit enhanced endothelial cell specificity

**Fig. 3:**
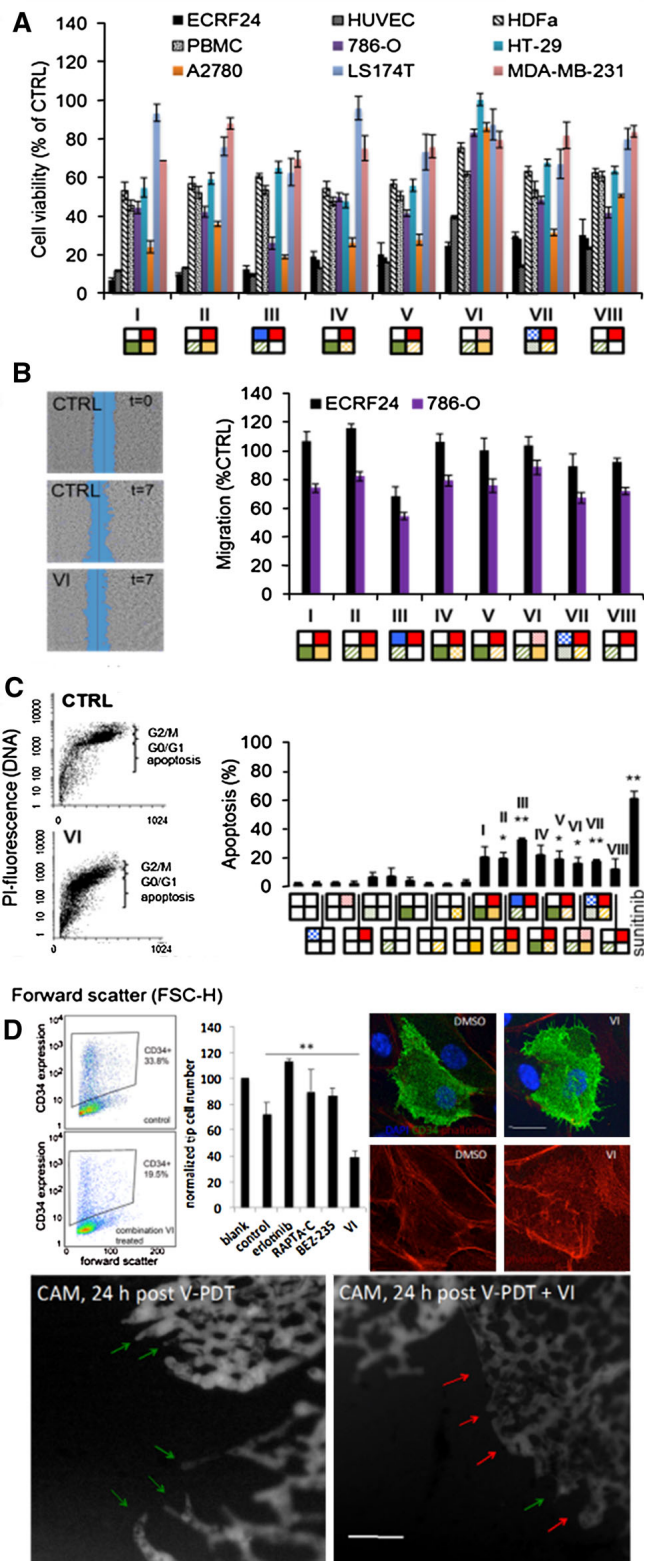
Validation of the best drug combinations. The effects of the most promising drug combinations (**I**–**VIII** from Fig. 2) were tested on the viability of the following non-malignant and cancerous cell lines: primary EC (HUVEC), adult human dermal fibroblasts (HDFa), human peripheral blood mononuclear cells (PBMCs) and five human tumor cell lines, i.e., A2780 ovarian adenocarcinoma, 786-O renal cell carcinoma, MDA-MB-231 breast adenocarcinoma and LS174T and HT-29 colorectal carcinomas (**a**) and on the migration of ECRF24 and 786-O cells (**b**). Images on the *left* show an example of migration assay, where a scratch is made in a cell monolayer at *t* = 0 and the relative closure of this scratch is measured after 7 h. **c** Effects of individual compounds and combinations on ECRF24 apoptosis induction. Images show the analysis of the DNA content by flow cytometry, after fixation of the cells in 70 % ethanol, a DNA extraction step and staining with PI for cells in the control (CTRL) and combination **VI** group. * indicates significance *p* value <0.05 and ** indicates significance *p* value <0.01 with student’s *t* test. Values represent the mean of at least two independent experiments with three replications each, and *error bars* represent the SEM. **d** Combination therapy **VI** inhibits tip cells in vitro and in vivo. FACS analysis show the decrease in CD34^+^ cells **VI** treated in HUVEC cultures, which is quantified in the bar graph and compared to single-drug treatments. CD34^+^ tip cells treated with **VI** present with a clearly different cellular organization of the actin fibers stained with phalloidin as compared to control cells, compatible with decreased migratory activity. *Bar* stands for 25 μm. The FITC-dextran fluorescence (FITC-dextran, 20 kDa, 20 μl, 25 mg/ml, Sigma-Aldrich) angiographies below show the chicken chorioallantoic membrane (CAM) capillary plexus at the edges of the Visudyne^®^-photodynamic therapy (V-PDT; 5 J/cm^2^ and 35 mW/cm^2^ at 420 ± 20 nm)-treated zones 24 h post-V-PDT, where the tip cells form the leading edge of the sprouting vasculature (*green arrows*). A major lack of sprouting tip cells (*red arrows*) is visible after treatment V-PDT with immediate of combination **VI**. (Color figure online)

The optimized drug combinations **I**–**VIII** (Fig. 2a) and corresponding single drugs were tested for viability of different cell types and shown in comparison with ECRF24 (Fig. 3a; Supplementary Fig. S5). The activity in ECRF24 was confirmed in primary ECs (HUVEC, Fig. 3a) and was much stronger than that of non-malignant cell types (adult human dermal fibroblasts (HDFa), human peripheral blood mononuclear cells (PBMCs) and tumor cells 786-O renal cell adenocarcinoma, HT-29 colorectal carcinomas, A2780 ovarian adenocarcinoma, LS174T colorectal adenocarcinoma and MDA-MB-231 breast adenocarcinoma), indicating an enhanced EC specificity. Combinations **I**–**VIII** only modestly affected cell motility in ECRF24 and 786-O cell lines in a wound-healing or scratch assay (Fig. 3b). The effect of individual compounds and combinations on ECRF24 apoptosis induction was assessed based on the analysis of the DNA content by flow cytometry. Several combinations (**II**, **III**, **V**, **VI**, **VII**) induced apoptosis in 20–30 % of ECRF24 cells (Fig. 3c).

Finally, the effect of combination **VI** on the inhibition of tip cells was assessed both in vitro and in vivo. FACS analysis shows a reduction in the number of CD34^+^ tip cells [32, 38, 39] after treating HUVEC cultures in vitro with **VI**. Quantification of these results is provided in the bar graph as compared to single-drug treatments (Fig. 3d, top left). Additionally, CD34^+^ tip cells treated with **VI** present with a clearly different cellular organization of the actin fibers stained with phalloidin, as compared to control cells (Fig. 3d, top right), compatible with decreased migratory activity.

The inhibition of tip cells in vivo was shown in the chicken chorioallantoic membrane (CAM) model following vaso-occlusive Visudyne^®^-photodynamic therapy (V-PDT) [33]. In the control treatment group, vascular sprouts led by tip cells can be seen growing into the treated area 24 h after V-PDT treatment starting the revascularization of the tissue (Fig. 3d, bottom left, green arrows). A significant reduction in the number of sprouting tip cells can be seen in the group treated with **VI** following V-PDT (Fig. 3d, bottom right, red arrows).

### Successful translation of optimal drug combinations into in vivo cancer models

**Fig. 4:**
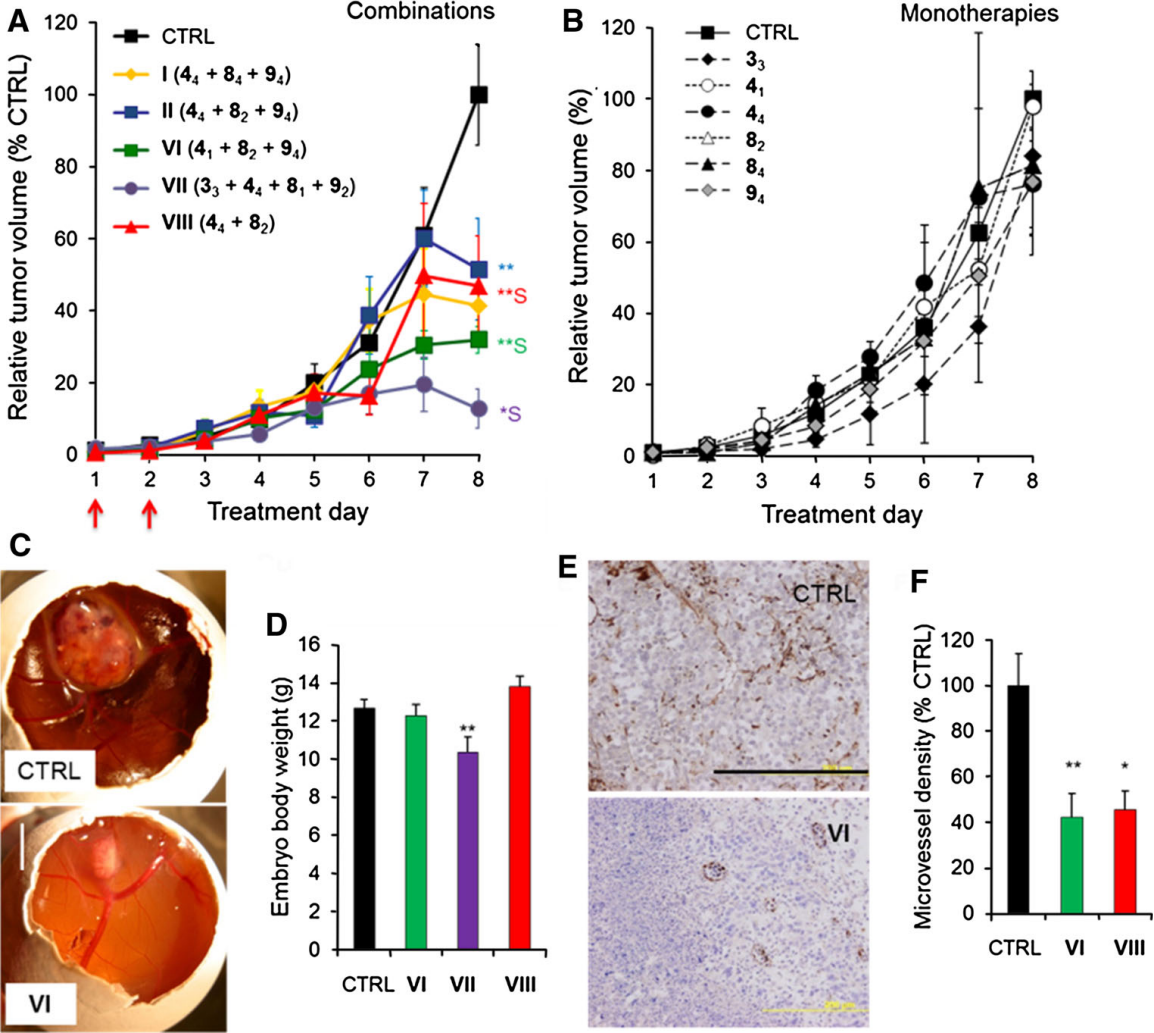
Inhibition of A2780 tumor growth on the CAM by the optimal drug combinations. Growth curves of A2780 tumors grafted on the CAM (*n* = 10) showing tumor volume as a function of treatment day for various drug combinations (**a**) and single-drug treatments (**b**). “*S*” indicates synergy. Compounds were freshly premixed and administered i.v. on treatment days 1 and 2 (*red arrows* in **a**). Data points represent the average tumor volume as a percentage of the final control volume per experiment. **c** Representative images of vehicle (CTRL) and combination **VI** treated tumors. **d** Mean embryo body weight on the last experiment day for selected treatment groups. **e** Representative images of CD31-stained tumor sections are shown. The *bar* in the lower right of the image represents 0.2 mm. The whole image was linearly adjusted for brightness and contrast. **f** Microvessel density quantification measured as the number of vessels per mm^2^ and represented as a percentage of the control (CTRL). * indicates *p* value <0.05 and ** indicates *p* value <0.01 student’s *t* test. *Error bars* represent the SEM. *N* = 3 for condition 4_1_. *N* = 5–11 for all other groups. (Color figure online)

A2780 cells were transplanted onto the chorioallantoic membrane of the chicken embryo and were allowed to grow forming vascularized tumors. Tumors were treated with combinations **I**, **II**, **VI**, **VII** and **VIII** by intravenous injection on treatment days 1 and 2 (Fig. 4a, red arrows). Doses (subsequently identified by a subscript) were translated to this model maintaining the drug dose ratios and taking into account the single-drug efficacy in this model (Fig. 4b; Table 2, Supplementary Methods). Drug combination **VII** (**3**_**3**_+**4**_**4**_+**8**_**1**_+**9**_**2**_) synergistically (CI 0.66) inhibited tumor growth by 87 % (* *p* < 0.03, Fig. 4a). Based on results in Fig. 3a, this activity could be due to the dual action on both ECRF24 and A2780 cells. Combination **VI** (**4**_**1**_+**8**_**2**_+**9**_**4**_) synergistically inhibited tumor growth by 68 % (Fig. 4a, c, **p < 0.002, CI 0.34) through mainly anti-angiogenic activity (compare Fig. 3a). Of note, none of these doses inhibited tumor growth significantly when applied individually (Fig. 4b). As group **VII** experienced weight loss (Fig. 4d, **p < 0.004), it was not further examined. Microvessel density (MVD) assessment (Fig. 4e) revealed that control tumors were well vascularized. MVD was 50–60 % lower in tumors treated with **VI** (**p < 0.008) and **VIII** (*p = 0.01, Fig. 4f). Based on these data, **VI** was selected as the most promising combination.

**Fig. 5:**
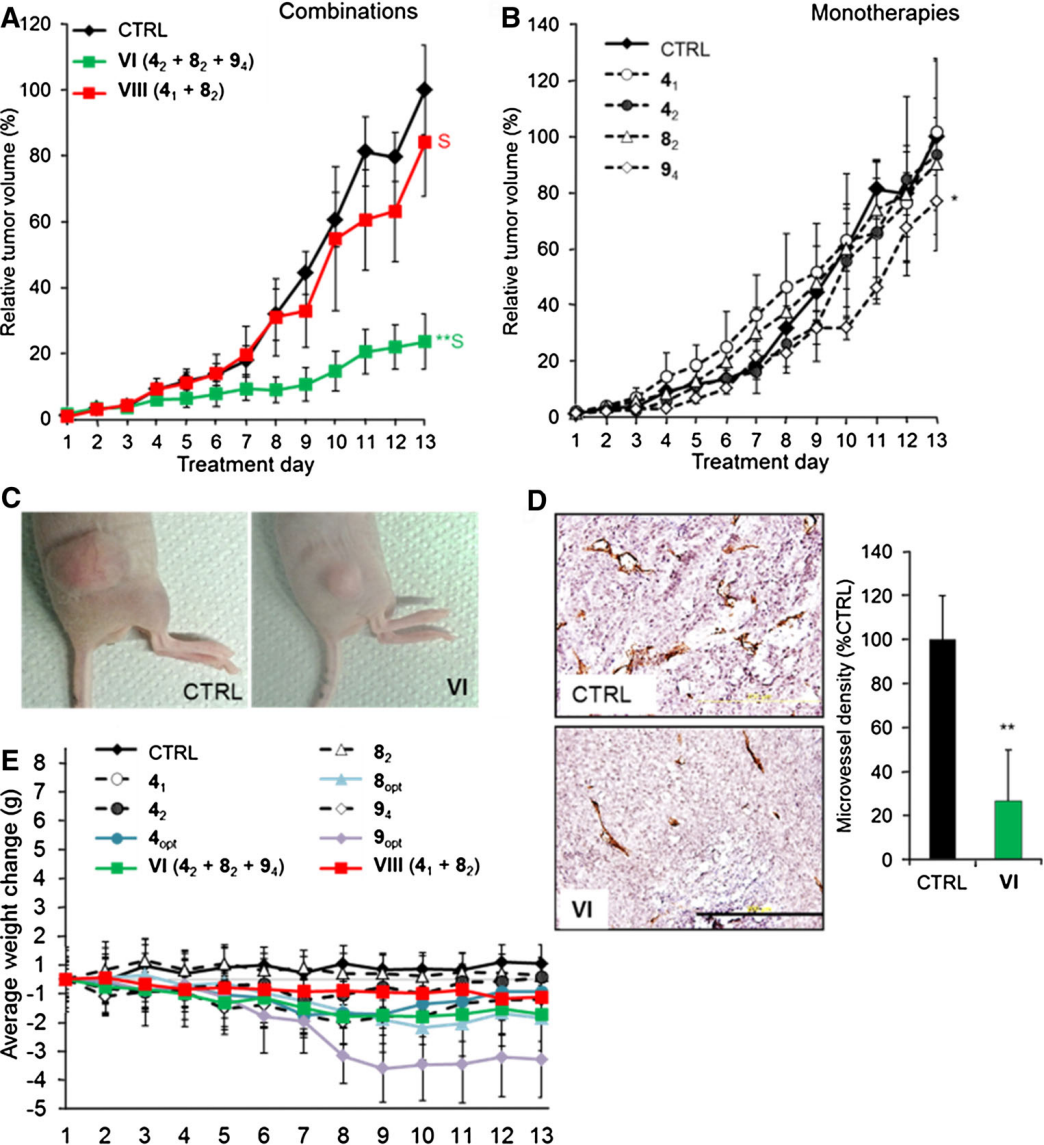
Inhibition of LS174T tumor growth in athymic mice by the optimal drug combinations. **a** LS174T tumors grafted subcutaneously in athymic Swiss nu/nu mice and treated daily with the drug combinations as listed in Table 2. **b** Inhibition of tumor growth by single compounds at indicated doses. *Data points* represent the average tumor volume as a percentage of the final CTRL volume per experiment, and *error bars* represent the SEM; *N* = 3–9. *p < 0.05 and **p < 0.01 (two-way ANOVA). “*S*” indicates synergy (CI < 1). **c** Representative images of vehicle-treated (CTRL) and tumors treated with drug combination **VI** on the last experiment day. **d** Representative images of immunohistochemical staining for the EC marker CD31 and corresponding quantification of microvessel density, measured as the number of vessels per mm^2^ and presented as a percentage of the CTRL. Results show significantly reduced microvessel density in tumors treated with **VI**. The *bar* in the *lower panel* image represents 0.2 mm and is valid for both images. The whole images were linearly adjusted for brightness and contrast. **e** Body weight change during the experiment. *p < 0.05 and **p < 0.01 (student *t* test). **4** (erlotinib), **8** (RAPTA-C) and **9** (BEZ-235). The *error bars* represent the SEM. (Color figure online)

Combinations **VI** and **VIII** were studied in athymic mice grafted with human LS174T colorectal adenocarcinoma. Drug doses were adapted to this model based on single-drug tumor growth inhibition efficacy (Supplementary Methods). Mice were treated daily with vehicle (CTRL), **VI** (**4**_**2**_+**8**_**2**_+**9**_**4**_) and **VIII** (**4**_**1**_+**8**_**2**_) (Fig. 5a; Table 2). **VI** and **VIII** inhibited tumor growth significantly by 76 ± 14 % (**p < 0.0001, CI 0.56) and 16 ± 16 % (CI 0.73), respectively (Fig. 5a). Drugs applied individually inhibited tumor growth only marginally, by 6 % (**4**_**2**_), 10 % (**8**_**2**_) or 23 % (**9**_**4**_) (*p < 0.013, Fig. 5b). Interestingly, since the LS174T cell line was not sensitive to **VI** (Fig. 3a), effective tumor inhibition (Fig. 5a, c) was attributed to the inhibition of angiogenesis. MVD assessment indicated that **VI** suppressed angiogenesis by approximately 80 % (**p < 0.001), compared with control tumors (Fig. 5d). No significant weight loss was recorded in either of the combinations tested (Fig. 5e). In contrast, individual compounds administered at optimal monotherapy doses, capable of effective tumor growth inhibition (Supplementary Fig. S6C) resulted in considerable body weight loss.

## Discussion

Next to the identification of novel targets as well as endogenous and synthetic novel angiogenesis inhibitors [40, 41], the combination of therapies is globally seen as a promising strategy to improve cancer therapy. The FSC technique was used to navigate through the large parametric space of nine compounds, each considered at four doses, aiming for an optimal angiostatic drug combination. Using a simple in vitro endothelial cell (EC) viability bioassay as the output, an optimal low-dose drug combinations containing axitinib (**3**), erlotinib (**4**), RAPTA-C (**8**) and BEZ-235 (**9**) was found. The most efficient of these combinations was also effectively inhibiting cancer in two in vivo animal models. We observed that (1) while some drugs showed synergistic interactions, others showed additive or even antagonistic behavior, (2) the observed synergy was drug dose ratio dependent, (3) the combination of angiostatic drugs enhanced endothelial specificity, (4) screening on EC migration did not identify highly efficient drug combinations, and (5) in vitro optimized anti-angiogenic drug combinations translated to anti-angiogenic anticancer effects in vivo.

We previously demonstrated that multi-drug effects can be expressed by a quadratic relationship of the drug–drug interactions [42], which was confirmed in bacterial systems [43]. Here, we have further demonstrated that the response surface for the whole range of drugs and drug doses applied can be expressed as a second-order equation that can be used to formulate optimal drug combinations. The results of this regression modeling (Supplementary Methods) permitted us to eliminate sunitinib (**6**) a compound which is known to have a similar target profile as axitinib (**3**) (note that both inhibit signaling of VEGF and PDGF [44]). The exclusion of sunitinib over axitinib appears justifiable, as axitinib is known to be a more selective TKI with stronger affinity for the same targets [44]. Similarly, the exclusion of bevacizumab (**2**) was expected, as it is known that EC does not use VEGF as an autocrine growth factor, and tumor angiogenesis is mainly driven by tumor produced VEGF [10].

The four drugs with significant inhibitory single-drug linear contributions to cell viability were compounds axitinib, erlotinib, RAPTA-C and BEZ-235 (Fig. 1d). In terms of intracellular signaling, this combination of drugs appears to make sense in retrospect. EGFR targeting by erlotinib and VEGFR targeting by axitinib result in inhibition of two largely synergistic and widely used cellular signaling pathways, i.e., the PI3K/AKT/mTOR and the ras/raf/MEK/MAPK signaling pathway, respectively. Since mTORC1 and mTORC2 belong to the PI3K/AKT pathway, one would expect that both signaling pathways are inhibited by EGFR and VEGFR inhibitors. It is also expected that a drug that targets histone proteins, such as RAPTA-C [45], can reinforce the angiostatic effect, as intervention with histone–DNA interactions is known to be angiostatic from the many reports on histone deacetylase inhibitors [46–48]. mTOR and EGFR inhibitors have already been identified as a synergistic combination in various cancer cell types [49, 50], despite clinically observed toxicity [51, 52].

Based on the analysis of the response surfaces of the second-order linear regression model generated from the four-drug optimization data (Fig. 2c) and embryo weight loss observed in the CAM model (Fig. 4d), axitinib was eliminated from further investigation. Thus, the optimal drug combination containing erlotinib, RAPTA-C and BEZ-235 was identified. It allowed for dose reductions of 5-, 11- and 6-fold, respectively, as compared to the equivalent single-drug dose efficiencies in vitro. Interestingly, enhanced EC specificity was observed for the combinations when compared to the individual compounds. This is another indication that the parallel blocking of multiple angiogenesis pathways can result in synergism for the angiostatic outcome. Simultaneous targeting of different signaling pathways may limit the probability of cells to develop acquired resistance [16].

The migration-based optimization screen failed to reach effective combinations (Supplementary Fig. S3B). This may suggest that proliferation is more dominant in the process of angiogenesis than cell migration, which has also been proposed by others [53]. The same may also be reflected by clinical trials, where proliferation inhibitors (such as sunitinib and BEZ-235) were more successful than migration inhibitors (the *α*_v_ *β*_3_ inhibitor cilengitide [54] and the *α*_5_ *β*_1_ antibody volociximab [55]). Another possible explanation for enhanced success with the proliferation assay over the migration assay may be the selective nature of synergistic drug interactions. As seen by Lehar et al. [15], drug combinations could attain greater selectivity. They suggested that “synergistic combinations tend to be more specific to particular cellular phenotypes than are single drugs.” This may explain the preferred success of the screen through selection on basis of proliferation, rather than cell migration.

The optimal drug mixture inhibited tumor growth by approximately 80 %, most likely by an inhibitory effect on angiogenesis. Although the detailed mechanism of combination therapy still needs to be understood, the induction of apoptosis as well as the inhibition of tip cells shows part of the effector mechanism. Targeting of tip cells may be another attractive strategy as these cells are indispensable for sprouting angiogenesis. The results provide a promising option for future clinical anti-angiogenic applications.

One might expect that the differences in pharmacokinetics between the components of the drug mixture may interfere with obtaining good results in vivo. Our results imply that (1) the best drug combinations found show smooth response surfaces (Fig. 2c, i.e., moderate changes in drug ratios do not significantly change the output), (2) response surfaces, giving a mathematical description of the magnitude of the interaction for all drug pairs, confirmed in vivo treatment outcome (compare Figs. 2c, 5a), and (3) EC viability observed in vitro seems to be a relatively good parameter for translation to vascular density reduction and tumor growth inhibition in vivo.

The current study shows that FSC applied in vitro can be used for the fast and reliable identification of potent, low-dose angiostatic drug combinations in vivo. It is likely that combining the optimal anti-angiogenic compounds with other treatment strategies may lead to even better cancer treatment outcomes. The capacity to normalize the tumor vasculature provides angiostatic strategies with outstanding combination therapy features [56, 57]. The impact of the method also lies in the fact that it can be applied in a variety of situations, e.g., for finding drug mixtures directly targeting tumor cells. This strategy would then also offer the opportunity for a personalized approach, by performing a drug screen on freshly isolated tumor cells from a patient biopsy. Such strategy would also depend on faster screening methods. We are currently working on improvement of the FSC method in order to make the selection procedure faster. In conclusion, designing effective, synergistic and specific multi-component drug combinations may become a key approach in developing new therapies for cancer and other diseases.

## Electronic supplementary material

Below is the link to the electronic supplementary material.

Supplementary material 1 (DOC 2982 kb)
