# Supplementary material for: Rapid optimization of drug combinations for the optimal angiostatic treatment of cancer
Source: Angiogenesis. 2015 Apr 1;18(3):233–44. doi: 10.1007/s10456-015-9462-9 (PMC4473022; doi:10.1007/s10456-015-9462-9)
Supplement: Supplementary file 1 — Supplementary material 1 (DOC 2982 kb) [file 10456_2015_9462_MOESM1_ESM.doc]

**Supplementary Material**

**Rapid optimization of drug combinations for the optimal angiostatic treatment of cancer**

Andrea Weiss^1,2^, Xianting Ding^3^, Judy R. van Beijnum^2^, Ieong Wong^4^, Tse J. Wong^2^, Robert H. Berndsen^1,2^, Olivier Dormond^5^, Marchien Dallinga^6^, Li Shen^7^, Reinier O. Schlingemann^6^, Roberto Pili^7^, Chih-Ming Ho^4^, Paul J. Dyson^1^, Hubert van den Bergh^1^, Arjan W. Griffioen^2^*, Patrycja Nowak-Sliwinska^1,2^

^1^Institute of Chemical Sciences and Engineering, Swiss Federal Institute of Technology, Lausanne, Switzerland. ^2^Department of Medical Oncology, VU University Medical Center, Amsterdam, The Netherlands. ^3^Med-X Research Institute, School of Biomedical Engineering, Shanghai Jiao Tong University, Shanghai, China. ^4^Department of Mechanical and Aerospace Engineering, University of California, Los Angeles, CA, USA. ^5^Department of Visceral Surgery, Centre Hospitalier Universitaire Vaudois, Lausanne, Switzerland. ^6^Department of Ophthalmology, Academic Medical Center, Amsterdam, The Netherlands. ^7^Department of Medicine, Roswell Park Cancer Institute, Buffalo, NY, USA.

Contents:

Supplementary Figures.

Supplementary Methods.

Supplementary References.

**Supplementary Figures.**

**Fig. S1.**

**
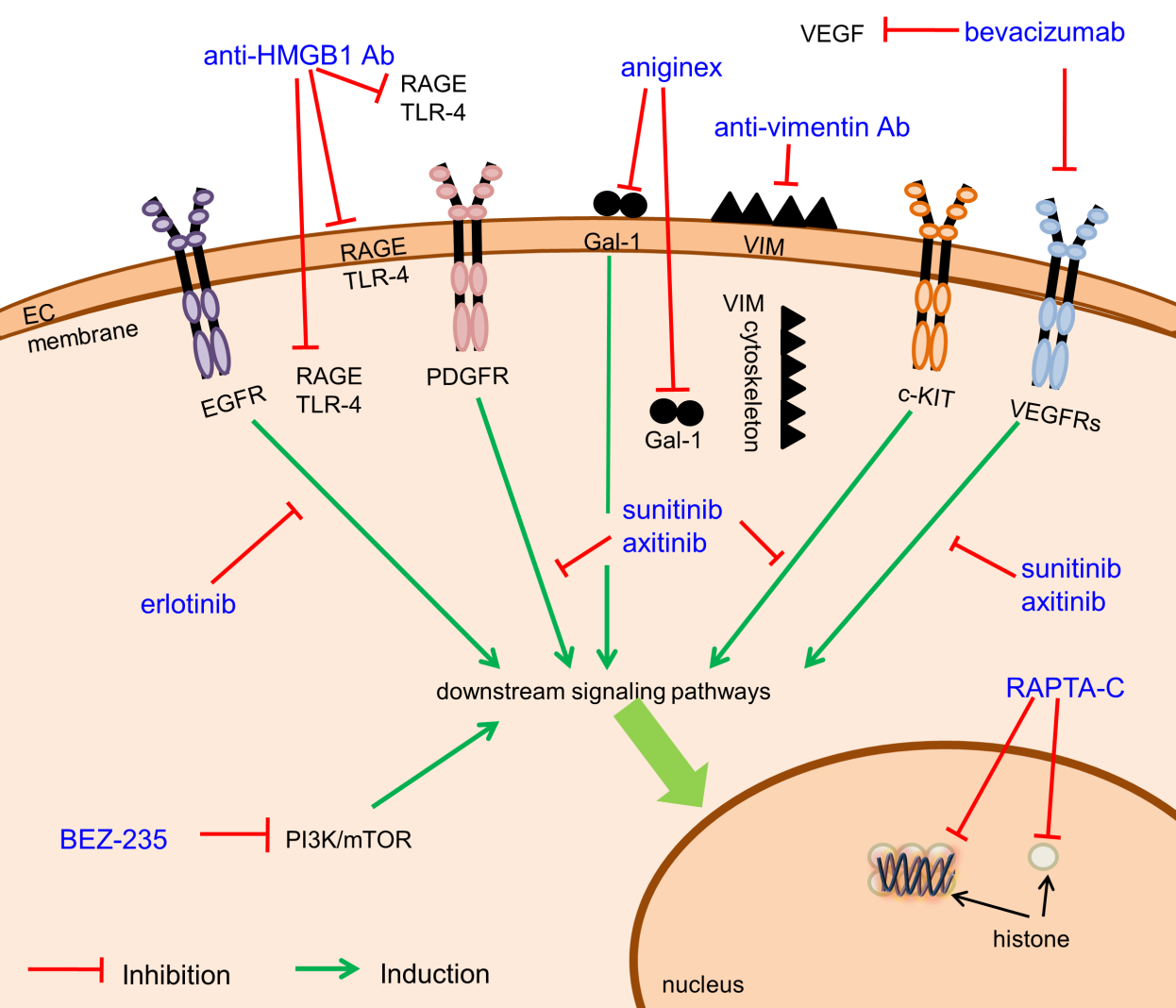
**

**Fig. S1.** Overview of drugs and targets. A schematic representation indicating where the nine angiostatic compounds included in the screen have their effect. EGFR, epidermal growth factor receptor; Gal-1, galectin-1; HMGB1, high mobility group box 1; PDGFR, platelet derived growth factor receptor; RAGE, receptor for advanced glycation end products; TLR-4, Toll like receptor 4; VEGF, vascular endothelial growth factor; VEGFRs, VEGF receptors; VIM, vimentin.

**Fig. S2.**

**
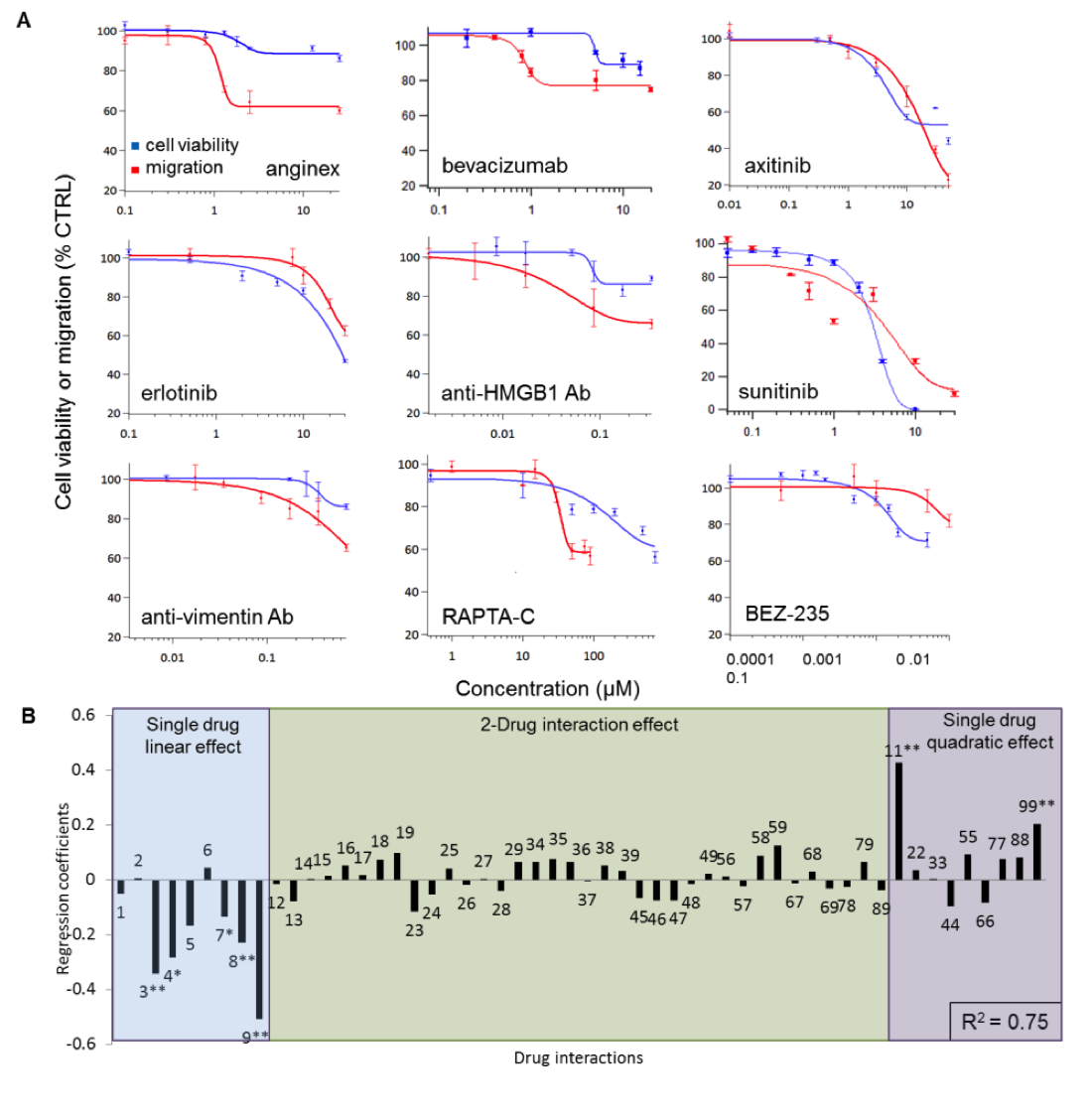
**

**Fig. S2.** Single drug dose-response curves and second-order linear regression model. (**A**) Results from single-drug assays on ECRF24 proliferation/viability (blue) and migration (scratch wound assay, red) performed over a large range of concentrations: anginex (**1**), bevacizumab (**2**), axitinib (**3**), erlotinib (**4**), anti-HMGB1 Ab (**5**), sunitinib (**6**), anti-vimentin Ab (**7**), RAPTA-C (**8**) and BEZ-235 (**9**). Each data point represents the average of at least two independent experiments, performed in triplicate. Error bars represent the SEM. (**B**) All regression coefficients of the linear regression model generated from the data obtained in the optimization of ECRF24 viability inhibition. The single drug linear effects of all compounds are presented in the left panel (blue), 2-drug interaction effects are presented in the middle panel (green), and single drug quadratic contributions are presented in the right panel (purple).

**Fig. S3.**


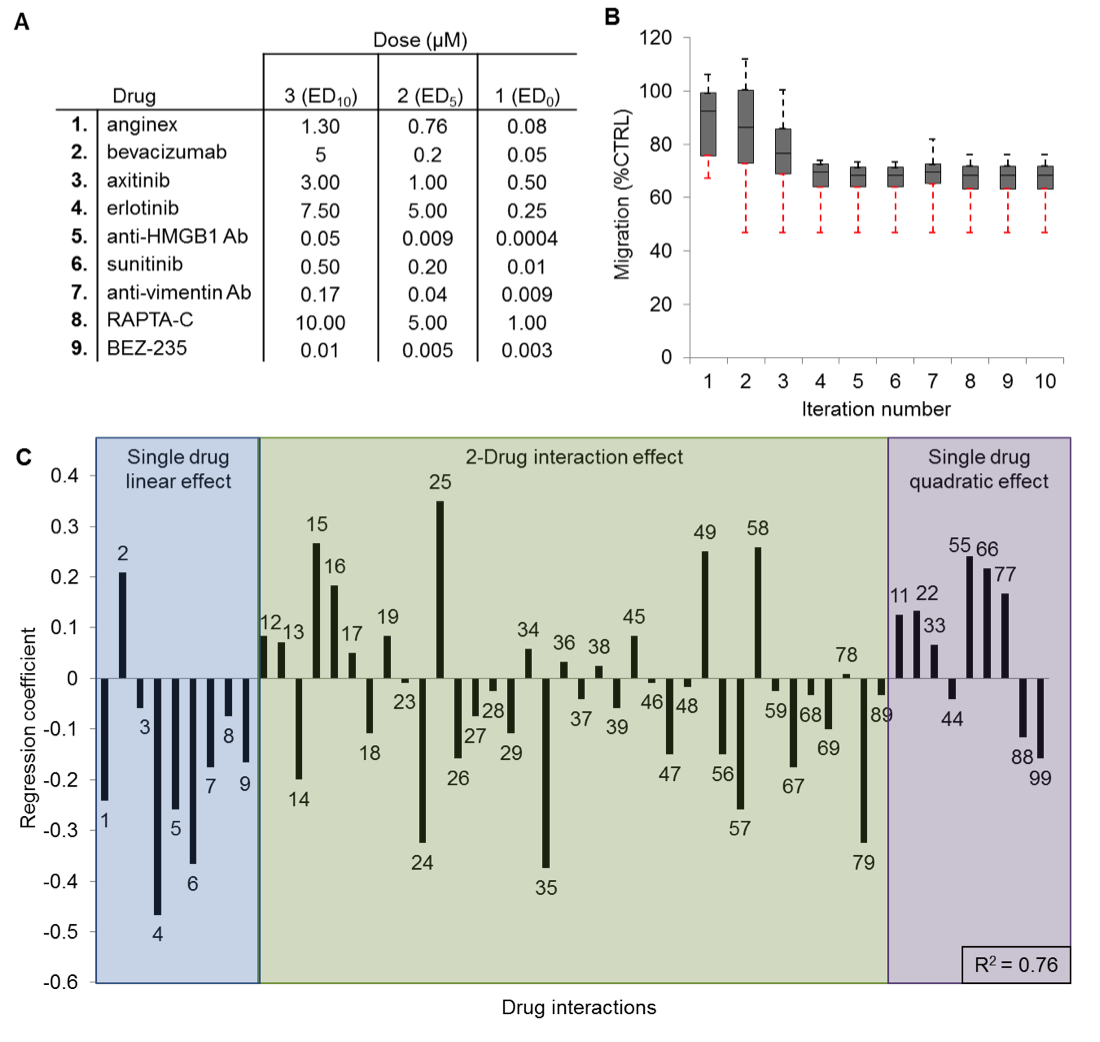


**Fig. S3.** FSC screen for optimal drug combinations based on migration inhibition. (**A**) The real drug doses represented by the coded doses “1”, “2” and “3” for each of the nine compounds used in the combination optimizations for migration inhibition. (**B**) Box-plot providing the range of the output values (*in vitro* ECRF24 migration, represented as percent of the control) for the most potent drug combinations identified by the end of each iterative cycle of the migration-based FSC optimization. The median and interquartile ranges of the outcome of the individual combinations are shown. Results show a slight decrease in output values followed by a plateau in the best cell output starting after iteration 4 (minimum values shown in red). (**C**) The regression coefficients obtained from the second-order linear regression model. The single drug linear contributions of all compounds are presented in the left blue panel, 2-drug interaction effects are presented in the middle green panel, and single drug quadratic contribution are presented in the right purple panel.

**Fig. S4.**

**
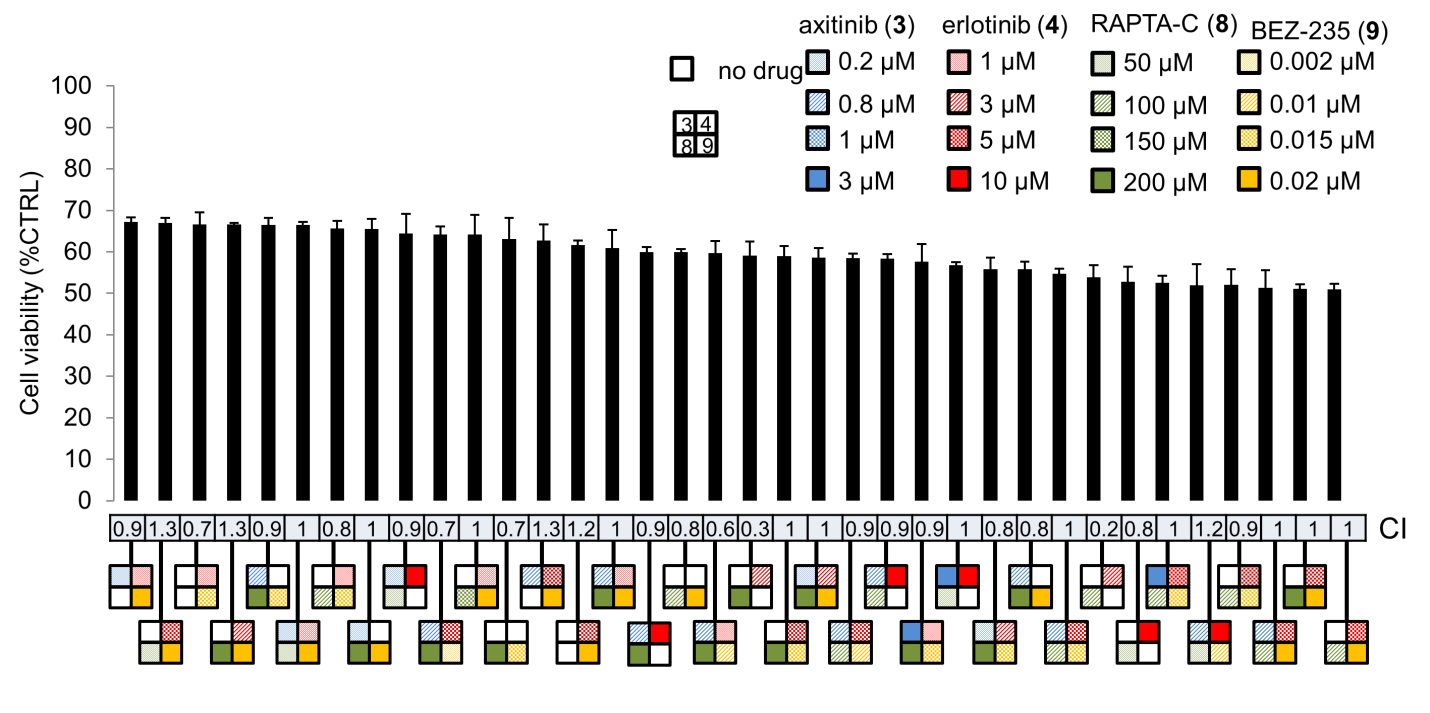
**

**Fig. S4.** Less efficient drug combinations tested in ECRF24 viability assay. The graph shows the combinations inhibiting the ECRF24 viability less than 50% with their corresponding CI values calculated using CompuSYN®, indicating synergistic (CI<1), additive (CI=1) or antagonistic (CI>1) drug combinations. The square icons present the specific combinations, where each position in the square and color corresponds to a specific drug and the filling pattern to the corresponding concentration: **3** (axitinib), **4** (erlotinib), **8** (RAPTA-C) and **9** (BEZ-235). The error bars represent the SEM.

**Fig. S5.**

**
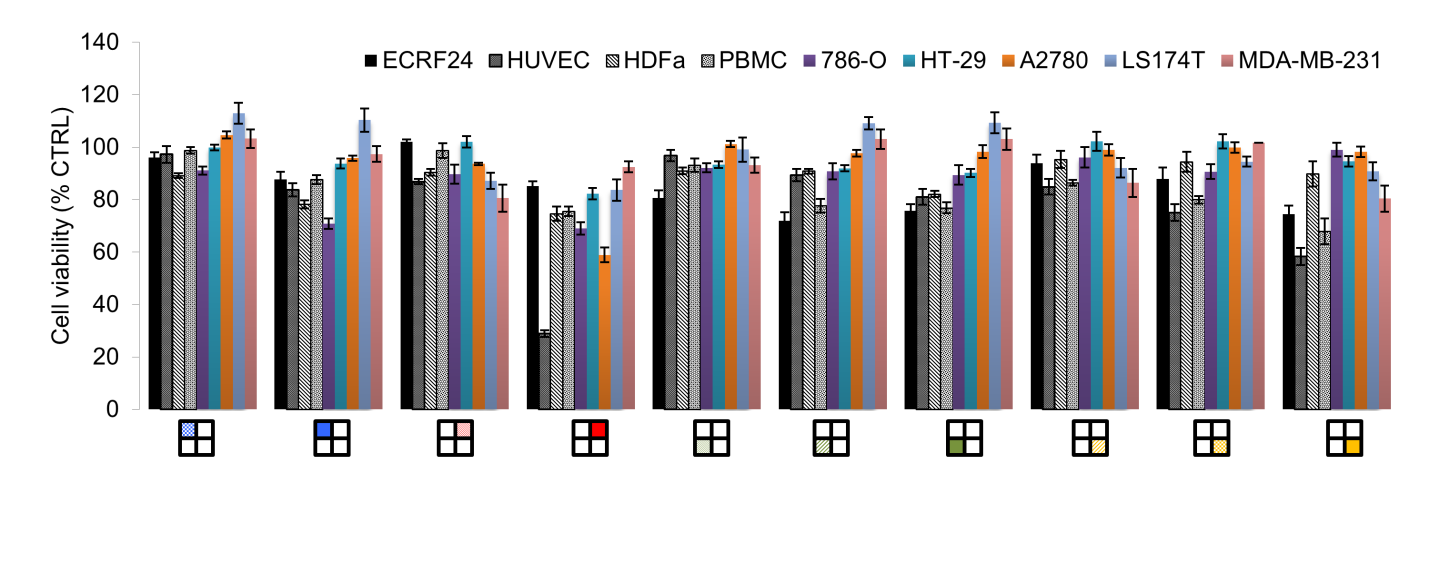
**

**Fig. S5.** The activity of individual drugs on viability of non-malignant and cancer cell types. The activity of drugs applied individually at the tested concentrations on various non-malignant and cancerous human cell types: immortalized human endothelial cells (ECRF24), human umbilical vein endothelial cells (HUVEC), adult human dermal fibroblasts (HDFa), human peripheral blood mononuclear cells (PBMC), ovarian adenocarcinoma (A2780), renal cell carcinoma (786-O), colorectal carcinoma (LS174T), breast adenocarcinoma (MDA-MB-231) and colorectal carcinoma (HT-29). Each data point represents the average of at least two independent experiments, performed in triplicate. Error bars represent the SEM.

**Fig. S6.**


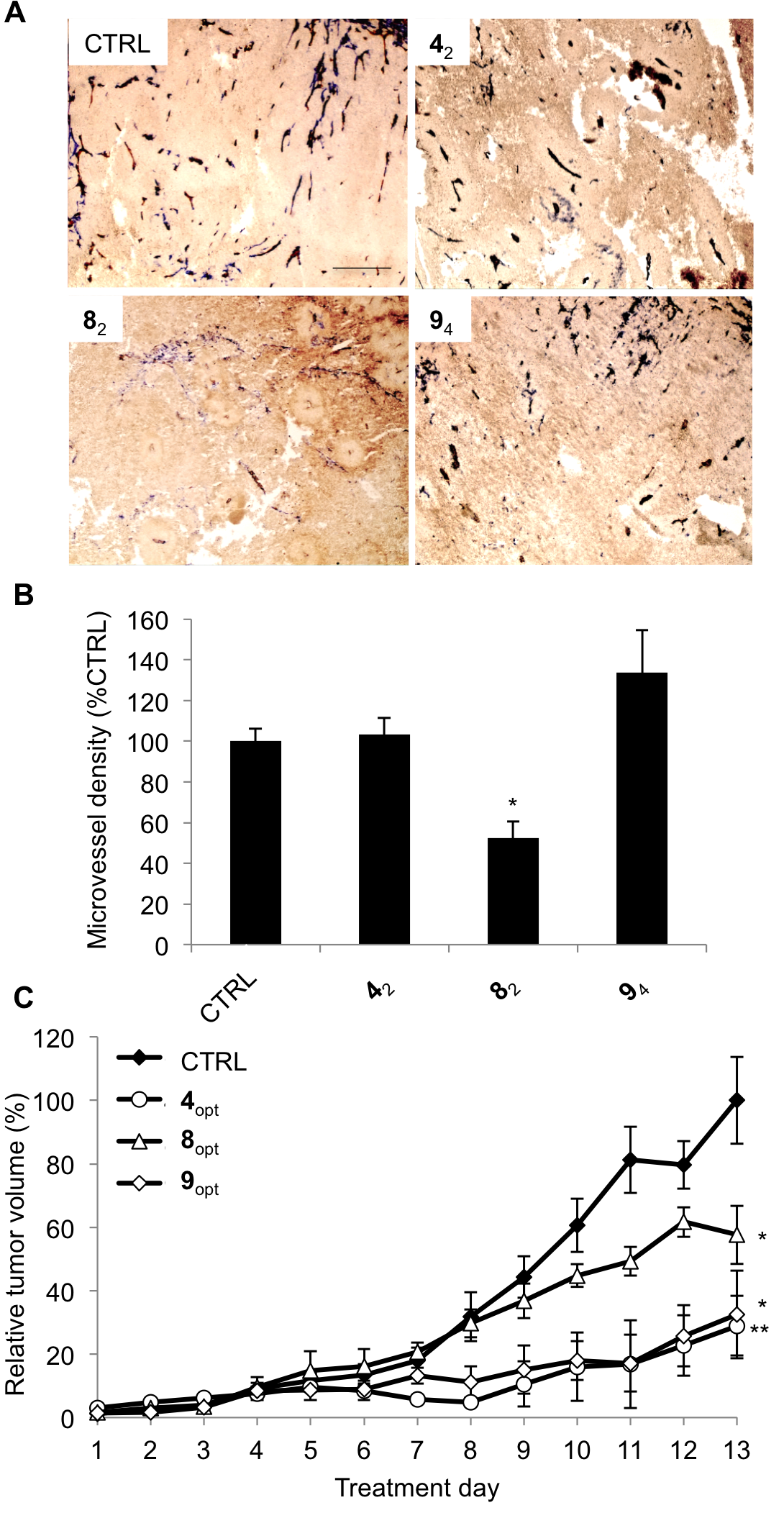


**Fig. S6.** Microvessel density and tumor growth curves of optimal single drug treatments. (**A**) CD31 positive IHC staining of mouse tumors treated with single drug therapies. The bar in the upper left image represents 0.5 mm and is valid for all images. (**B**) Corresponding quantification of microvessel density represented as a percent of the control. (**C**) Effect of single drug therapy on tumor growth at high concentrations (**4**_opt_ = 50 mg/kg; **8**_opt_ = 100 mg/kg, **9**_opt_ = 30 mg/kg). N_treated_ = 2-3; N_CTRL_ = 9; *p < 0.05; **p < 0.01. Error bars represent the SEM. **4** (erlotinib), **8** (RAPTA-C) and **9** (BEZ-235).

**Supplementary Methods**

**Drug acquisition**

Axitinib and erlotinib were purchased from LC laboratories (Woburn, MA, USA), Sutent® (sunitinib) from Pfizer Inc. (New York, NY, USA) and BEZ-235 from Chemdea LLC (Ridgewood, USA). RAPTA-C was synthesized and purified as described previously. Avastin® (bevacizumab) was obtained from Genentech (San Francisco, CA, USA). Anti-vimentin monoclonal mouse antibody (clone V9) was purchased from Dako (Glostrup, Denmark) and anti-HMG1 antibody from Santa Cruz Biotechnology (Heidelberg, Germany). Anginex® was provided by PepTx (Excelsior, MN, USA).

**Drug selection justification**

The set of nine drugs included in this screen contains compounds targeting a broad spectrum of signaling pathways. Three tyrosine kinase inhibitors were included, i.e. sunitinib (**6**), axitinib (**3**) and erlotinib (**4**). Sunitinib/Sutent® is a broad-spectrum TKI known to inhibit the receptors of vascular endothelial growth factor (VEGFR-1, -2, and -3), as well as the platelet-derived growth factor (PDGFR-β), mast/stem cell growth factor (c-KIT) and the fibroblast growth factor (FGFR-1). Axitinib/Inlyta® is a second generation TKI with fewer targets (the VEGFR’s, PDGFR-β, and c-KIT) but with a relatively higher affinity for its targets than sunitinib . Sunitinib and axitinib were included in this screen due to their extensive clinical use in the treatment of various cancer types; i.e. pancreatic neuroendocrine tumors , renal cell carcinoma (RCC) , gastrointestinal stromal tumors for sunitinib . Erlotinib/Tarceva® is a small molecule EGFR inhibitor. The EGFR is highly expressed in a wide spectrum of tumors, including RCC, head and neck- and breast carcinoma. EGFR overexpression plays a significant role in tumor growth and progression, including the promotion of proliferation, invasion and metastasis. Part of its effects are also through the inhibition of angiogenesis . We selected erlotinib due to its different target kinases (HER1/EGFR), as compared to sunitinib or axitinib.

We also included three anti-angiogenic antibodies, i.e. bevacizumab (**2**), anti-HMGB1 (**5**) and anti-vimentin (**7**). Bevacizumab/Avastin®, a humanized monoclonal antibody which targets and neutralizes circulating VEGF , was the first clinically approved angiogenesis inhibitor. Bevacizumab is approved for the treatment of RCC in combination with interferon-alpha , breast cancer , glioblastomas and lung cancer . There may be debate over its use in our *in vitro* screen as endothelium produced VEGF may not contribute to tumor angiogenesis. The anti-vimentin antibody, targeting extracellular vimentin, exerts potent anti-angiogenic and anti-tumor activity *in vivo* . *In vitro* endothelial cells are extremely sensitive to vimentin targeting. Vimentin has been shown to be overexpressed in the endothelium of various cancer types . The anti-HMGB1 (high-mobility group box 1) antibody targets a molecule known to act as a pro-inflammatory and pro-angiogenic cytokine. We have recently shown that the targeting of HMGB1 has a promising anti-cancer effect, as it represents an angiostatic and anti-inflammatory strategy .

Anginex (**1**) is a beta-sheet-forming 33-mer designer peptide targeting galectin-1. It has shown specific angiostatic and tumor growth inhibitory activity . This specific angiogenesis inhibitor acts directly on tumor ECs. RAPTA-C (**8**) is an experimentally designed and validated ruthenium-based anti-metastatic metal-organic compound with angiostatic properties. Although its mechanism of action is not fully understood, a recent study has indicated that RAPTA-C acts, at least in part, through histone binding.

Finally, we included BEZ-235/Dactolisib (**9**), a dual PI3K/mTOR1/2 inhibitor. It has shown efficacy in various cancer types. mTOR is a serine/threonine kinase that controls numerous cellular activities through the function of two main protein complexes, mTORC1 and mTORC2 . mTORC1 regulates the expression and stability of HIF-1 along with key proteins involved in EC proliferation. mTORC2 is implicated in the regulation of cell morphology and adhesion, as well as being responsible for the phosphorylation and activation of the Akt oncogene, a potent stimulator of angiogenesis and tumorogenesis . PI3K and mTOR are frequently co-expressed in cancer cells and their overexpression has been correlated with decreased survival in patients .

It is clear that the above selected compounds are not the only possible selection of anti-angiogenic compounds for the present purposes. However, we believe that the first search among the compounds that are known to be effective in tumor anti-angiogenesis is not an unreasonable approach. The compounds that have no anti-tumor activity as single compounds can nevertheless help or interfere with cancer growth in combination with other drugs.. It is not necessary to have the detailed knowledge about the drug selected, as the knowledge is never complete, as shown by the fact that antagonism may well show up (CI>1).

**Cell culture and maintenance**

Immortalized human vascular endothelial cells (ECRF24) were maintained in medium containing 50% DMEM and 50% RPMI 1640 supplemented with 1% of antibiotics (Life Technologies, Carlsbad, California, USA). ECRF24 were always cultured on 0.2% gelatin coated surfaces. A2780 cells (human ovarian carcinoma) were maintained in RPMI 1640, supplemented as described above. Adult human dermal fibroblast (HDFa), 786-O (renal cell adenocarcinoma), HT-29 (colorectal adenocarcinoma), LS174T (colon adenocarcinoma) and MDA-MD-231 (breast adenocarcinoma) cells were maintained in DMEM, supplemented as mentioned above. Human umbilical vein ECs (HUVECs) were isolated and cultured as previously described. Human peripheral blood mononuclear cells (PBMC) were freshly isolated as previously described.

**Dilutions and culture conditions**

Drug mixing was performed as follows: stock solutions were first used to prepare the highest concentration of each compound and lower concentrations were prepared through serial dilutions of the higher concentrations. All drug concentrations were prepared 9 times more concentrated than desired to account for dilution by other compounds (or medium when a compound was not included). The drug combinations were prepared directly before applying drug combinations to cells by first adding the required amount of medium for each combination and then adding the required concentration of each compound, always in the same order.

**The Feedback System Control (FSC) technique and data modeling**

In order to design an effective anti-cancer therapy containing multiple drugs it is necessary to deal with control of non-linear complex networks that are most likely not fully characterized. Various approaches were employed previously to optimize drug combinations . These methods include empirical and non-systematic approaches based on clinical experience with individual drugs , as well as modern alternative methods . The latter include five different approaches:

(i) Techniques using exhaustive tests trying all possible drug combinations using a high-throughput screening technology . Most studies, however, use only pairs of drugs , but the extension of this technique to a larger number of drugs in the combination seems to be difficult.

(ii) Statistical approaches based on linear combinations of known input-output relations and desired phenotype, neglecting non-linearities in biological networks. These models treat the system as a black box and do not rely on a complete characterization of the biological networks .

(iii) Model-based combinations where biological measurements are used to build explicit models of a target network using simulations . This class seems to be an essential approach in a successful multi drug design, however, it should be noted that complete knowledge on all drugs and their targets is not available yet.

(iv) Model-free biological search algorithms, where drugs are iteratively combined and measured, searching the huge search space and using the black box approach, similarly to (ii) . At each iteration a high-throughput assay measures cellular (or organism) phenotype in response to a drug combination, which is then again inserted into the algorithm. Here, the algorithm generates new candidate combinations to be tested, based on the previous results. These methods use e.g. the Gur-Game algorithm , or a genetic algorithm . The key difference between the methods (ii) and (iii) is that statistical model-based techniques attempt to approximate the control landscape using training data and then optimize the approximated response, while the model-free methods typically optimize the control landscape on a discretized grid without explicitly constructing a model.

(v) Model and biological search algorithm, i.e. an approach integrating (ii, iii and iv). Here, model-based predictions of effective perturbations are combined with a closed-loop iterative experimental search .

The limitation of modeling cell behavior lies in the complexity of the cellular signaling pathways and, in the case of cancer, its inter-patient and intratumoral heterogeneity . Although it is possible to make generalized observations about cell mechanisms and their interactions based on mathematical models, these models are inherently constrained by the information given to the system and the assumptions used in generating the models. Systematic based searches are also limited due to the number of possible existing drug combinations and therefore by the time, cost and effort required to test drug combinations.

The advantage of the Feedback System Control (FSC) technique, belonging to approach (iv) of the approaches listed above, is that it is phenotypically driven and, as such, does not require any prior mechanistic information, such as complex cellular signaling information or target identification. This allows rapid converging upon an experimentally verifiable optimal drug combination . FSC uses differential evolution (DE) and is based on integrative system responses, where the difference between desired and real system responses are used as optimization criteria to be fed into a search algorithm, which can then iteratively drive the system to a desired systemic fate . In addition, the DE algorithm implements parallel searches allowing for fast identification of optimal drug combinations.

For many biological applications, the FSC scheme is ideal because it requires no knowledge of the mechanisms involved in determining the cellular response to a given drug stimulus or input. It only requires an output value that describes the overall cellular activity in response to a drug combination. This output is fed into the closed-loop feedback system, in order for the search algorithm to determine the next iteration of drug combinations to be tested on the cell system.

A cellular response was selected in order to quantify the activity of each drug combination tested *in vitro* during the FSC process. This allows definition of the overall anti-angiogenic potential of the optimal drug combination. Selection of the biological test to create the output is very important. Endothelial cell migration and viability are required in the process of angiogenesis . Therefore, we selected *in vitro* assays for the inhibition of these processes as indicators of the inhibition of angiogenesis.

All data points from the optimizations were fitted to Matlab-based linear regression and stepwise linear regression models. These models describe the response surface of the cells in terms of the bioassay output. Response surfaces were used to quantify the contribution of each drug to the inhibition of the system’s outputs and to identify the interactions between different drugs. The accuracy and robustness of these models were assessed through two methods: the fitted accuracy (R^2^) and the predictive potential of the models. Plotting the experimental data vs. the fitted data and determining the R^2^ value assessed the fitted accuracy. This value indicates how well the experimental and fitted data are correlated. Subsequently, we used the linear regression model in order to predict highly effective drug combinations. We obtained a relatively good predictive power for the model when combinations were tested *in vitro* (data not shown). Appropriate data and model verification methods were implemented to ensure the accuracy and reliability of model-based predictions. The main assumptions of linear regression models were verified, i.e. weak exogeneity, linearity, constant variance, independence of errors, and lack of multicolinearity. As the presence of multicolinearity was indicated in a few of the regressors in the second-order linear regression model on the cell viability data (based on the analysis of variance inflation factors and condition indices), a stepwise linear regression was also performed to isolate the most important regressors and remove instabilities in the regression analysis due to multicolinearity.

The second-order linear regression model represents a mathematical description of the relationship between the individual drug combinations as input and the corresponding output values, i.e. experimentally measured cell viability. For a multi-drug cancer cell system, a complete second-order linear regression model would be in the following form:


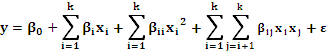


where β_0_, β_i_, β_ii_ and β_ij_  are the intercept, linear, quadratic and bilinear (or interaction) terms, respectively; γ is the response variable (in our case, the cell proliferation or cell migration efficacy); x_i_ and x_j_ are independent variables (in our case, the drugs); ε is an error term with a mean equal to zero .

A common purpose to investigate the second-order linear regression model is to quantify causality between independent variables (x_i_ and x_j_) and response variables (γ) with a high level of scientific inference. When the data is drawn from reasonably homogeneous populations, the relationship between various independent variables and the mean of the distribution for a response variable can often be predicted with high fidelity.

In our study, we built second-order linear regression models to examine the relationships between independent variables (drugs) and response variables (cell proliferation or cell migration). Furthermore, single drug linear effects (β_i_), 2-drug interaction effects (β_ij_) and single drug quadratic effects (β_ii_) are analyzed to facilitate our decision for effective drug combinations.

Negative values of the second-order term regression coefficients correspond to synergistic effects, whereas positive values correspond to antagonistic effects. Appropriate data and model verification methods were implemented to ensure the accuracy and reliability of predictions made based on these models. The main assumptions of linear regression models were verified (i.e. weak exogeneity, linearity, constant variance, independence of errors, and lack of multicolinearity). As the presence of multicolinearity was indicated in a few of the regressors in the second-order linear regression model of the cell viability data (based on the analysis of variance inflation factors and condition indices), a stepwise linear regression was also performed to isolate the most important regressors and remove instabilities in the regression analysis due to multicolinearity. Drug interactions were additionally analyzed using the CompuSYN software , where a combination index (CI) is calculated for each drug combinations, where CI values less than 1 indicate synergistic drug combinations, CI-values greater than 1 indicate antagonistic drug combinations, and a CI equal to one represents an additive effect.

**Apoptosis assay and assessment of tip cell characteristics.**

Apoptosis was measured by assessment of sub-diploid cells. After drug exposure, cells were detached and incubated in buffer containing 2.5 µM citric acid, 45 µM Na_2_HPO_4_ and 0.1% Triton-X100, (pH 7.4, 20 min, 37°C) to extract small molecular weight DNA. Cells were subsequently analyzed with a FACSCalibur (BD Biosciences) in the FL2 channel and apoptotic cells were defined as having less DNA than cells in G1 phase of cell cycle.

Tip cells can be identified in vitro by staining for CD34 . After drug exposure, cell suspensions were obtained by TrypLE (Gibco) treatment of adherent endothelial cell monolayers. All immunofluorescent labeling and washing was performed in PBS containing 0.1% bovine serum albumin. Cells were fixed in 2% paraformaldehyde in PBS for 15 min at room temperature and incubated with anti-CD34-phycoerythrin (anti-CD34-PE; clone QBend-10, Dako). Cells were analyzed on a FACSCalibur flow cytometer (Becton Dickinson, Franklin Lakes, NJ, USA) in combination with FlowJo 6.4.7 software (Tree Star, San Carlos, CA, USA). Non-stained and non-treated cells were used as controls. Maximum DMSO concentration in cultures was 0.28% and displayed no measurable activity in cell assays.

Tip cells were visualized in the chorioallantoic membrane (CAM) of fertilized chicken embryos . Twenty four hours after inducing angiogenesis by closure of the vasculature through photodynamic therapy (5 J/cm^2^, with irradiance of 35 mW/cm^2^ at 420 ± 20 nm, drug-light interval 1 min) with Visudyne® (Novartis Pharma Inc., Hettlingen, Switzerland) as a photosensitizer (V-PDT), tip cell mediated sprouting angiogenesis occurs at the borders of the treated area. Tip cell activity was visualized (epi-fluorescence microscope, Nikon AG, Eclipse E 600 FN, Japan) in CAMs treated (V-PDT + **VI**) or not (V-PDT) with drug mixture **VI**, by injection of FITC-dextran (20 kDA, 20 μl, 25 mg/ml, Sigma-Aldrich, St. Louis, USA), as well as 20 μl of India ink (Pelikan,Witzikon, Switzerland) administered into extra-embryonic cavity in order to enhance vascular contrast.

**Colorectal carcinoma xenograft model.**

All experiments were carried out in female 7-week old athymic Swiss mice (Charles River), according to a protocol approved by the Committee for Animal Experiments for the Canton Vaud, Switzerland (license 2736). Mice were treated daily by oral gavage (erlotinib and BEZ-235) or i.p. injection (RAPTA-C) in a 100 µl volume, or vehicle only for control mice (see Table 2). It is therefore likely that pharmacokinetics and pharmacodynamics vary between the drugs and that the dose ratios established *in vitro* may not be translatable to mice. The single drug doses required for combinations in the murine model were therefore established directly by testing in animals. We realize that *in vitro*, ECs are continuously exposed while *in vivo* exposure may be intermittent, based on the T_1/2_, which diverges from the *in vitro* system. T_1/2_ values *in vivo* for individual drugs are known, i.e. 36 h for erlotinib, 9 h for RAPTA-C, and 12 h for BEZ-235 . We tested single drugs at 2-3 doses (5 mice per group) using the protocol described in the Materials and Methods section. Doses to be used in combination were selected based on this preliminary data. At the end of experiments, mice were euthanized and tumors were resected and fixed in zinc fixative solution for immunohistochemistry.

**Immunohistochemistry**

5 µm sections were blocked with 5% BSA in PBS followed by incubation with primary antibodies against CD31 (1:200; clone SZ31, Dianova, Hamburg, Germany). Secondary donkey anti-rat biotinylated antibodies (1:200; Jackson, Suffolk, UK) were then incubated, followed by Streptavidin-HRP (1:50; Dako, Glostrup, Denmark), and visualized by 3,3'-diaminobenzidine (DAB).

**Supplementary References**

1. Allardyce CS, Dyson PJ, Ellis DJ, Heath SL. [Ru(eta(6)-p-cymene)Cl-2(pta)] (pta=1,3,5-triaza-7-phosphatricyclo[3.3.1.1]decane): a water soluble compound that exhibits pH dependent DNA binding providing selectivity for diseased cells. Chem Commun. 2001:1396-7.

2. Bukowski R. Third generation tyrosine kinase inhibitors and their development in advanced renal cell carcinoma. Front Oncol. 2012;2:13.

3. Kim S, Ding W, Zhang L, Tian W, Chen S. Clinical response to sunitinib as a multitargeted tyrosine-kinase inhibitor (TKI) in solid cancers: a review of clinical trials. Onco Targets Ther. 2014;7:719-28.

4. Jonasch E, Signorovitch JE, Lin PL, Liu Z, Culver K, Pal SK, et al. Treatment patterns in metastatic renal cell carcinoma: a retrospective review of medical records from US community oncology practices. Current medical research and opinion. 2014:1-10.

5. Lee-Ying R, Lester R, Heng DY. Current management and future perspectives of metastatic renal cell carcinoma. International journal of urology : official journal of the Japanese Urological Association. 2014.

6. Nishida T, Doi T, Naito Y. Tyrosine kinase inhibitors in treatment of unresectable or metastatic gastrointestinal stromal tumors. Expert opinion on pharmacotherapy. 2014:1-11.

7. Ling J, Johnson KA, Miao Z, Rakhit A, Pantze MP, Hamilton M, et al. Metabolism and excretion of erlotinib, a small molecule inhibitor of epidermal growth factor receptor tyrosine kinase, in healthy male volunteers. Drug Metab Dispos. 2006;34:420-6.

8. Presta LG, Chen H, O'Connor SJ, Chisholm V, Meng YG, Krummen L, et al. Humanization of an anti-vascular endothelial growth factor monoclonal antibody for the therapy of solid tumors and other disorders. Cancer research. 1997;57:4593-9.

9. Escudier B, Pluzanska A, Koralewski P, Ravaud A, Bracarda S, Szczylik C, et al. Bevacizumab plus interferon alfa-2a for treatment of metastatic renal cell carcinoma: a randomised, double-blind phase III trial. Lancet. 2007;370:2103-11.

10. Kumler I, Christiansen OG, Nielsen DL. A systematic review of bevacizumab efficacy in breast cancer. Cancer treatment reviews. 2014.

11. Brandes AA, Mason W, Pichler J, Nowak AK, Gil M, Saran F, et al. Can bevacizumab prolong survival for glioblastoma patients through multiple lines of therapy? Future oncology. 2014;10:1137-45.

12. Koyama N. Adverse cardiovascular events predict survival benefit in non-small lung cancer patients treated with bevacizumab. Cancer biomarkers : section A of Disease markers. 2014;14:259-65.

13. Mukohara T, Engelman JA, Hanna NH, Yeap BY, Kobayashi S, Lindeman N, et al. Differential effects of gefitinib and cetuximab on non-small-cell lung cancers bearing epidermal growth factor receptor mutations. J Natl Cancer Inst. 2005;97:1185-94.

14. Thompson EW, Paik S, Brunner N, Sommers CL, Zugmaier G, Clarke R, et al. Association of increased basement membrane invasiveness with absence of estrogen receptor and expression of vimentin in human breast cancer cell lines. Journal of cellular physiology. 1992;150:534-44.

15. Hu L, Lau SH, Tzang CH, Wen JM, Wang W, Xie D, et al. Association of Vimentin overexpression and hepatocellular carcinoma metastasis. Oncogene. 2004;23:298-302.

16. van Beijnum JR, Nowak-Sliwinska P, van den Boezem E, Hautvast P, Buurman WA, Griffioen AW. Tumor angiogenesis is enforced by autocrine regulation of high-mobility group box 1. Oncogene. 2013;17:363-74.

17. Dings RP, Chen X, Hellebrekers DM, van Eijk LI, Zhang Y, Hoye TR, et al. Design of nonpeptidic topomimetics of antiangiogenic proteins with antitumor activities. J Natl Cancer Inst. 2006;98:932-6.

18. Nowak-Sliwinska P, van Beijnum JR, Casini A, Nazarov AA, Wagnieres G, van den Bergh H, et al. Organometallic ruthenium(II) arene compounds with antiangiogenic activity. J Med Chem. 2011;54:3895-902.

19. Rugo HS, Stopeck A, Joy AA. A randomized, double-blind phase II study of the oral tyrosine kinase inhibitor (TKI) axitinib (AG-013736) in combination with docetaxel (DOC) compared to DOC plus placebo (PL)

in metastatic breast cancer (MBC). J Clin Oncol. 2007;25(18 supl):32.

20. Adhireksan Z, Davey GE, Campomanes P, Groessl M, Clavel CM, Yu H, et al. A ligand distinctionbetween two ruthenium-cymen anticancer agents determines protein versus DNA targeting. Nature Communications. 2014;5:3462.

21. Sabatini DM. mTOR and cancer: insights into a complex relationship. Nature reviews Cancer. 2006;6:729-34.

22. Benjamin D, Colombi M, Moroni C, Hall MN. Rapamycin passes the torch: a new generation of mTOR inhibitors. Nature reviews Drug discovery. 2011;10:868-80.

23. Corradetti MN, Guan KL. Upstream of the mammalian target of rapamycin: do all roads pass through mTOR? Oncogene. 2006;25:6347-60.

24. Wu P, Liu T, Hu Y. PI3K inhibitors for cancer therapy: what has been achieved so far? Curr Med Chem. 2009;16:916-30.

25. Zhou S, Liu L, Li H, Eilers G, Kuang Y, Shi S, et al. Multipoint targeting of the PI3K/mTOR pathway in mesothelioma. Br J Cancer. 2014;110:2479-88.

26. Elfiky AA, Aziz SA, Conrad PJ, Siddiqui S, Hackl W, Maira M, et al. Characterization and targeting of phosphatidylinositol-3 kinase (PI3K) and mammalian target of rapamycin (mTOR) in renal cell cancer. J Transl Med. 2011;9:133.

27. van Beijnum JR, Dings RP, van der Linden E, Zwaans BM, Ramaekers FC, Mayo KH, et al. Gene expression of tumor angiogenesis dissected: specific targeting of colon cancer angiogenic vasculature. Blood. 2006;108:2339-48.

28. Boyum A. Isolation of lymphocytes, granulocytes and macrophages. Scandinavian journal of immunology. 1976;Suppl 5:9-15.

29. Feala JD, Cortes J, Duxbury PM, Piermarocchi C, McCulloch AD, Paternostro G. Systems approaches and algorithms for discovery of combinatorial therapies. Wiley interdisciplinary reviews Systems biology and medicine. 2010;2:181-93.

30. Dancey JE, Chen HX. Strategies for optimizing combinations of molecularly targeted anticancer agents. Nature reviews Drug discovery. 2006;5:649-59.

31. Park M, Nassar M, Vikalo H. Bayesian active learning for drug combinations. IEEE transactions on bio-medical engineering. 2013;60:3248-55.

32. Decker S, Sausville EA. Preclinical modeling of combination treatments: fantasy or requirement? Annals of the New York Academy of Sciences. 2005;1059:61-9.

33. Tol J, Koopman M, Cats A, Rodenburg CJ, Creemers GJ, Schrama JG, et al. Chemotherapy, bevacizumab, and cetuximab in metastatic colorectal cancer. N Engl J Med. 2009;360:563-72.

34. Jia J, Zhu F, Ma X, Cao Z, Li Y, Chen YZ. Mechanisms of drug combinations: interaction and network perspectives. Nature reviews Drug discovery. 2009;8:111-28.

35. Calzolari D, Bruschi S, Coquin L, Schofield J, Feala JD, Reed JC, et al. Search algorithms as a framework for the optimization of drug combinations. PLoS computational biology. 2008;4:e1000249.

36. Zimmermann GR, Lehar J, Keith CT. Multi-target therapeutics: when the whole is greater than the sum of the parts. Drug discovery today. 2007;12:34-42.

37. Greco WR, Bravo G, Parsons JC. The search for synergy: a critical review from a response surface perspective. Pharmacological reviews. 1995;47:331-85.

38. Borisy AA, Elliott PJ, Hurst NW, Lee MS, Lehar J, Price ER, et al. Systematic discovery of multicomponent therapeutics. Proceedings of the National Academy of Sciences of the United States of America. 2003;100:7977-82.

39. Lamb J, Crawford ED, Peck D, Modell JW, Blat IC, Wrobel MJ, et al. The Connectivity Map: using gene-expression signatures to connect small molecules, genes, and disease. Science. 2006;313:1929-35.

40. Potti A, Dressman HK, Bild A, Riedel RF, Chan G, Sayer R, et al. Genomic signatures to guide the use of chemotherapeutics. Nature medicine. 2006;12:1294-300.

41. Lehar J, Zimmermann GR, Krueger AS, Molnar RA, Ledell JT, Heilbut AM, et al. Chemical combination effects predict connectivity in biological systems. Molecular systems biology. 2007;3:80.

42. Nelander S, Wang W, Nilsson B, She QB, Pratilas C, Rosen N, et al. Models from experiments: combinatorial drug perturbations of cancer cells. Molecular systems biology. 2008;4:216.

43. Sun CP, Usui T, Yu F, Al-Shyoukh I, Shamma J, Sun R, et al. Integrative systems control approach for reactivating Kaposi's sarcoma-associated herpesvirus (KSHV) with combinatory drugs. Integr Biol (Camb). 2009;1:123-30.

44. Wong PK, Yu F, Shahangian A, Cheng G, Sun R, Ho CM. Closed-loop control of cellular functions using combinatory drugs guided by a stochastic search algorithm. Proceedings of the National Academy of Sciences of the United States of America. 2008;105:5105-10.

45. Yoon BJ. Enhanced stochastic optimization algorithm for finding effective multi-target therapeutics. BMC Bioinformatics. 2011;12 Suppl 1:S18.

46. Zinner RG, Barrett BL, Popova E, Damien P, Volgin AY, Gelovani JG, et al. Algorithmic guided screening of drug combinations of arbitrary size for activity against cancer cells. Mol Cancer Ther. 2009;8:521-32.

47. Zhao B, Hemann MT, Lauffenburger DA. Intratumor heterogeneity alters most effective drugs in designed combinations. Proceedings of the National Academy of Sciences of the United States of America. 2014;111:10773-8.

48. Price KV, Lampinen JA, Storn RM, SpringerLink (Online service). Differential Evolution A Practical Approach to Global Optimization. Natural computing series,. Berlin, Heidelberg: Springer-Verlag Berlin Heidelberg; 2005.

49. Ho DH, C.M. System Control-Mediated Drug Delivery Towards Complex Systems via Nanodiamond Carriers. International Journal of Smart and Nano Materials. 2010;1:70-81

50. Griffioen AW, Molema G. Angiogenesis: potentials for pharmacologic intervention in the treatment of cancer, cardiovascular diseases, and chronic inflammation. Pharmacological reviews. 2000;52:237-68.

51. Yoon H, Lee JY, Suk HJ, Lee S, Lee H, Lee S, et al. Modeling to predict growth/no growth boundaries and kinetic behavior of salmonella on cutting board surfaces. Journal of food protection. 2012;75:2116-21.

52. Van der Borght K, Van Craenenbroeck E, Lecocq P, Van Houtte M, Van Kerckhove B, Bacheler L, et al. Cross-validated stepwise regression for identification of novel non-nucleoside reverse transcriptase inhibitor resistance associated mutations. BMC bioinformatics. 2011;12:386.

53. Seber GAF, Lee AJ. Linear Regression Analysis; 2nd Edition: Wiley; 2003.

54. Chou TC, Martin N. CompuSyn for Drug Combinations: PC Software and User’s Guide: A Computer Program for Quantitation of Synergism and Antagonism in Drug Combinations, and the Determination of IC50 and ED50 and LD50 Values. NJ: Paramus; 2005.

55. Siemerink MJ, Klaassen I, Vogels IM, Griffioen AW, Van Noorden CJ, Schlingemann RO. CD34 marks angiogenic tip cells in human vascular endothelial cell cultures. Angiogenesis. 2012;15:151-63.

56. Nowak-Sliwinska P, Segura T, Iruela-Arispe ML. The chicken chorioallantoic membrane model in biology, medicine and bioengineering. Angiogenesis. 2014;17:779-804.

57. Nowak-Sliwinska P, van Beijnum JR, van Berkel M, van den Bergh H, Griffioen AW. Vascular regrowth following photodynamic therapy in the chicken embryo chorioallantoic membrane. Angiogenesis. 2010;13:281-92.

58. Lu JF, Eppler SM, Wolf J, Hamilton M, Rakhit A, Bruno R, et al. Clinical pharmacokinetics of erlotinib in patients with solid tumors and exposure-safety relationship in patients with non-small cell lung cancer. Clinical pharmacology and therapeutics. 2006;80:136-45.

59. Scolaro C, Bergamo A, Brescacin L, Delfino R, Cocchietto M, Laurenczy G, et al. In vitro and in vivo evaluation of ruthenium(II)-arene PTA complexes. J Med Chem. 2005;48:4161-71.
